# Se‐Assisted Modulation of Electronic Structure of Ruthenium Phosphide Nanotubes for Efficient Alkaline Hydrogen Evolution Reaction

**DOI:** 10.1002/smsc.202400610

**Published:** 2025-03-13

**Authors:** Yongju Hong, Eunsoo Lee, Jae Hun Seol, Tae Kyung Lee, Songa Choi, Seong Chan Cho, Taekyung Kim, Hionsuck Baik, Sangyeon Jeong, Sung Jong Yoo, Sang Uck Lee, Kwangyeol Lee

**Affiliations:** ^1^ Department of Chemistry and Research Institute for Natural Sciences Korea University Seoul 02841 Republic of Korea; ^2^ Hydrogen·Fuel Cell Research Center Korea Institute of Science and Technology (KIST) Seoul 02792 Republic of Korea; ^3^ School of Chemical Engineering Sungkyunkwan University Suwon 16419 Republic of Korea; ^4^ Korea Basic Science Institute (KBSI) Seoul 02841 Republic of Korea; ^5^ Division of Energy & Environment Technology KIST School University of Science and Technology (UST) Daejeon 34113 Republic of Korea; ^6^ KHU‐KIST Department of Converging Science and Technology Kyung Hee University Seoul 02447 Republic of Korea

**Keywords:** alkaline water electrolysis, cation‐exchange, doping, electronic structures, hydrogen evolution reactions, phosphides

## Abstract

Anion‐exchange membrane water electrolysis (AEMWE) holds immense promise for hydrogen (H_2_) production yet faces challenges due to the sluggish kinetics of the hydrogen evolution reaction (HER). Highly efficient and durable catalysts for HER are crucial for the successful implementation of AEMWE to produce hydrogen gas reliably. Ruthenium phosphides (Ru_*x*_P) have emerged as promising non‐Pt catalysts for alkaline HER; however, they suffer from rapid degradation due to weak Ru—P bonding, which cannot protect the Ru center from further oxidation and subsequent dissolution. Herein, first‐principles calculations indicate the enhanced stability of Ru—Se against oxidation compared to Ru—P, highlighting the importance of introducing Se into the Ru_2_P phase. Electrochemical studies using the selenium (Se)‐doped Ru_2_P double‐walled nanotubes (Ru_2_(P_0.9_Se_0.1_) DWNTs) demonstrate significantly lower overpotentials (29 mV @ 10 mA cm^−2^) and robust stability (>50 h) in 1.0 m KOH, surpassing those of Pt/C. In AEMWE, Ru_2_(P_0.9_Se_0.1_) DWNTs exhibit an outstanding performance (10.31 A cm^−2^ @ 80 °C, stable @ 1.0 A cm^−2^ for ≈200 h), surpassing state‐of‐the‐art catalysts. The findings of this study highlight the pivotal role of anion modification in enhancing the catalytic stability and performance for efficient hydrogen production in AEMWE systems.

## Graphical abstract

Selenium‐doped ruthenium phosphide (Ru_2_(P_0.9_Se_0.1_)) is obtained via ion‐engineering techniques. Ru sites in Ru_2_(P_0.9_Se_0.1_) act as primary catalytic centers, optimizing intermediate adsorption energies. The precisely controlled Se doping enhances the Ru−OH moieties formation on the surface while preventing Ru dissolution. This catalyst demonstrates outstanding cell performances, indicating the promise of anion‐modified Ru_2_P for efficient hydrogen evolution in anion‐exchange membrane water electrolysis.

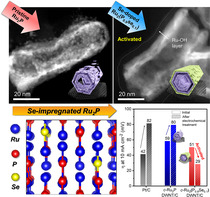

## 1 Introduction

Anion‐exchange membrane water electrolysis (AEMWE) technology has recently achieved high hydrogen (H_2_) production purity and harbors significant economic potential.^[^ ^1^ ^]^ However, its efficiency is markedly impeded by the sluggish kinetics of the hydrogen evolution reaction (HER), primarily due to the lack of catalytic systems that boost both H_2_O dissociation and the conversion of H* into H_2_ in alkaline media. Metal phosphide systems have been actively studied as promising non‐Pt catalyst candidate for alkaline HER due to their high electrical conductivity, low overpotential, and low cost.^[^ ^2^, ^3^, ^4^, ^5^, ^6^, ^7^ ^]^ Among the various metal phosphides, ruthenium phosphides (Ru_*x*_P) have demonstrated excellent catalytic activity toward alkaline HER,^[^ ^8^, ^9^, ^10^, ^11^, ^12^ ^]^ which could be essential for the broad success of AEMWE systems.^[^ ^13^, ^14^, ^15^ ^]^ However, Ru_*x*_P catalysts typically suffers from rapid surface transformation and loss of their initial performance, owing to the inevitable formation of active oxidized species on the surface, ease of solvation, and subsequent dissolution by the electrolyte, which undermines the structural integrity.^[^ ^10^, ^16^, ^17^ ^]^ Therefore, it is urgent to develop a Ru_*x*_P material system that can form and maintain robust and active catalytic sites to enable the practical operation of AEMWE, which requires atomic‐level surface structure engineering of catalysts.

A straightforward strategy to fine‐tune the electronic and coordination behavior of surface Ru species is to replace some of the P matrix anions with other heteroanions. The incorporation of heteroanions into ionic compounds can lead to a catalytic performance comparable to that of Pt‐ or Ir‐based catalysts, rendering them promising as cost‐effective catalysts.^[^ ^18^, ^19^, ^20^, ^21^ ^]^ The different sizes and charges of heteroanions would greatly affect the coordination behaviors of the catalytic Ru centers, which could improve the HER performance by fine‐tuning the binding of intermediates (H_2_O, OH, and H species) and by adjusting the oxygen adsorption energy and lattice cohesive energy of Ru_2_P.^[^ ^22^, ^23^, ^24^, ^25^ ^]^ Furthermore, the lattice distortion caused by embedding heteroatoms into the Ru_2_P coordination environment could enhance the resistance of Ru to solvation and dissolution. First‐principles calculations indicated that Ru—Se possesses significantly higher cohesive energy relative to Ru—P against lattice degradation caused by surface oxidation. Simultaneously, the Se doping strategy ensured the preservation of the Ru–OH moiety as an active oxidized species on the catalyst surface, resulting in accelerated HER kinetics in alkaline media. However, this effect is dependent on the careful regulation of the Se dopant concentrations, emphasizing the need for precise control over the doping levels to achieve the desired outcome.^[^ ^26^, ^27^, ^28^ ^]^

Here, we demonstrate a straightforward method for the fabrication of Ru_2_P double‐walled nanotubes (DWNTs) followed by Se incorporation. This process entails the partial substitution of Ru^3+^ ions into the overlayer of Cu_3−*x*_P–S single‐walled nanotubes (CPS SWNTs), followed by Se‐exchange to form well‐defined crystalline (*c*‐) Ru_2_(P_0.9_Se_0.1_) DWNTs. The catalytic performance of the synthesized *c*‐Ru_2_(P_0.9_Se_0.1_) DWNTs catalysts was extensively investigated and compared with those of other Ru‐based catalysts and commercial Pt catalysts. Our combined experimental investigation revealed that Se doping within the Ru_2_P matrix resulted in a strong interaction between Ru and Se, markedly enhanced the stability as the active Ru–OH species forms on the catalyst surface. The exceptional performance of the *c*‐Ru_2_(P_0.9_Se_0.1_) DWNTs was attributed to the presence of the Ru–OH moiety on the *c*‐Ru_2_(P_0.9_Se_0.1_) DWNTs catalysts, causing a significant downshift in the *d*‐band center of the Ru sites. This alteration in the electronic structure, specific to the *c*‐Ru_2_(P_0.9_Se_0.1_) DWNTs catalysts, further optimized the binding of H* intermediates, thereby further enhancing H_2_ production (Tafel step).

The successful integration of Se into the Ru_2_P phase significantly accelerated the kinetics of the overall alkaline HER (Volmer and Tafel step) and improved the resistance to dissolution. As a result, the electrochemically activated *c*‐Ru_2_(P_0.9_Se_0.1_) DWNTs catalysts exhibited a significantly low overpotential of 29 mV at a current density of 10 mA cm^−2^ in 1.0 m KOH, as well as a robust stability for over 50 h under alkaline HER conditions, surpassing the performance of Pt/C catalysts. Furthermore, when utilized into AEMWE as the cathode catalyst, the *c*‐Ru_2_(P_0.9_Se_0.1_) DWNTs achieved a remarkable performance of 10.31 A cm^−2^ at 80 °C and stable operation under 1.0 A cm^−2^ for ≈200 h. This high current density reaction produced by the *c*‐Ru_2_(P_0.9_Se_0.1_) DWNTs catalyst holds significant implications for large‐scale hydrogen production in industrial applications, outperforming state‐of‐the‐art catalysts in AEMWEs.

## 2 Result

### 2.1 Formation Mechanism and Characterization of Crystalline Ru_2_(P_0.9_Se_0.1_) DWNTs

**Figure 1:**
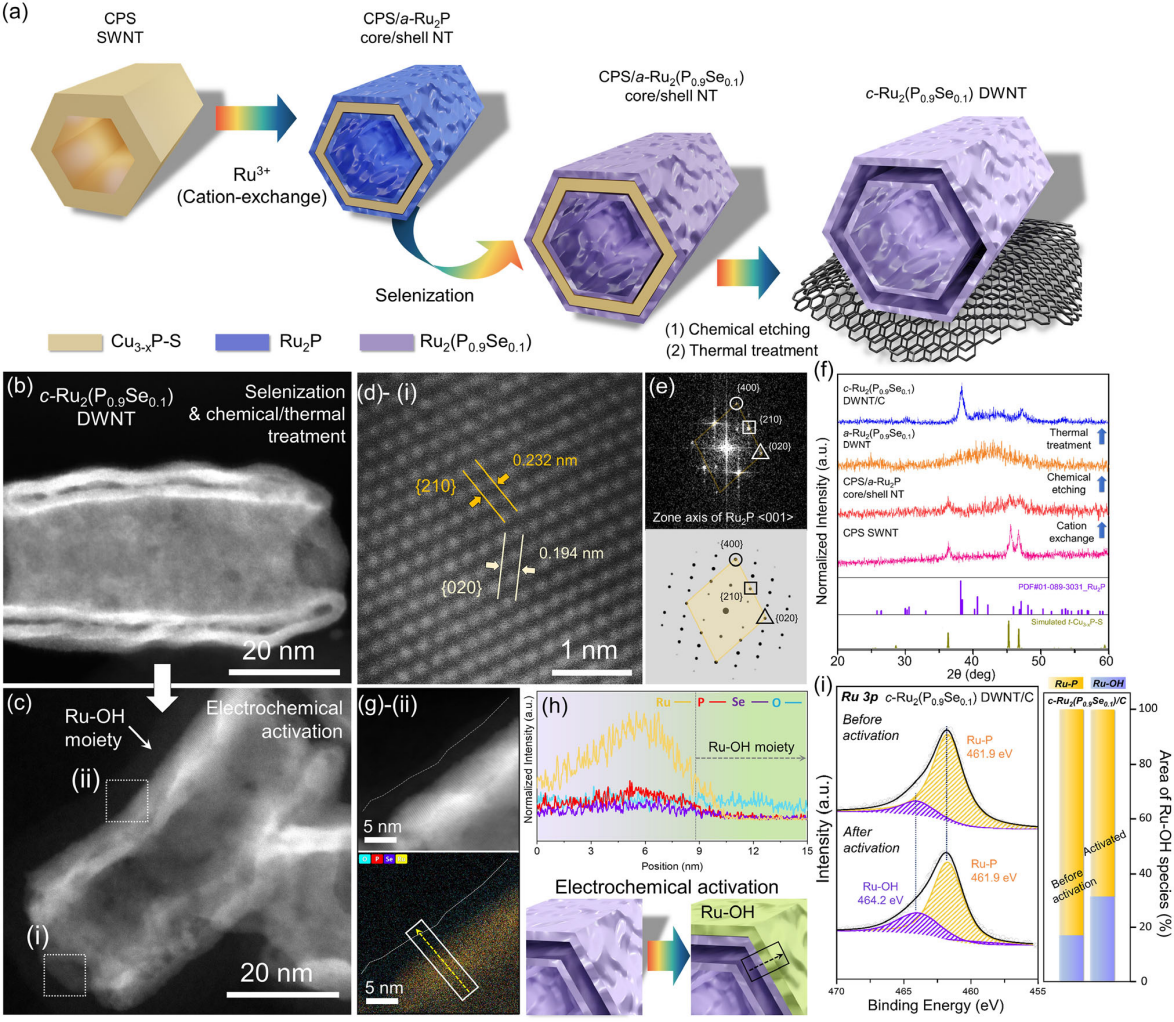
Synthesis and structural characterization of *c*‐Ru_2_(P_0.9_Se_0.1_) DWNTs before and after electrochemical activation. a) Schematic illustration of the synthesis process for *c*‐Ru_2_(P_0.9_Se_0.1_) DWNTs utilizing anion/partial cation‐exchange reaction from CPS SWNTs, followed by chemical and thermal treatment. b,c) HAADF‐STEM images of *c‐*Ru_2_(P_0.9_Se_0.1_) DWNTs before and after electrochemical activation. d) Atomic‐resolution HAADF‐STEM image focusing on area (i) in (c). e) FFT pattern of activated *c*‐Ru_2_(P_0.9_Se_0.1_) DWNTs alongside corresponding simulated FFT patterns of Ru_2_P phase. f) XRD patterns of nanotubes undergoing structural evolution from CPS SWNTs to *c*‐Ru_2_(P_0.9_Se_0.1_) DWNTs. g) HAADF‐STEM images of activated *c‐*Ru_2_(P_0.9_Se_0.1_) DWNTs from the area (ii) in (c) with combined elemental mapping image. h) Line profiling analysis along the direction shown in (g). i) Ru 3*p* XPS of *c*‐Ru_2_(P_0.9_Se_0.1_) DWNT/C before and after electrochemical activation. Corresponding area percentage of deconvoluted Ru 3*p* XPS of c‐Ru_2_(P_0.9_Se_0.1_) DWNT/C.

The formation of crystalline Se‐doped Ru_2_P DWNTs (*c*‐Ru_2_(P_0.9_Se_0.1_) DWNTs) is presented in **Figure** **1**a. Initially, the uniform sixfold CPS SWNTs were prepared from Cu_1.81_S nanorods (CS NRs) as a cation‐exchange platform with average height and width of 92.3 ± 2.5 and 60.6 ± 2.0 nm, respectively, using a previously established method (Figure S1–S3, Supporting Information).^[^ ^29^, ^30^ ^]^ Subsequently, two‐step cation (Ru)‐ and anion (Se)‐exchange reactions were conducted, resulting in the transformation of the outermost layers of the CPS SWNTs and the incorporation of Se, which led to the formation of a core/shell structure composed of CPS and amorphous (*a*‐) Ru_2_(P_0.9_Se_0.1_) nanotubes (core/shell CPS/*a‐*Ru_2_(P_0.9_Se_0.1_) NTs) (Figure S4a–c, Supporting Information).^[^ ^31^ ^]^ Notably, the Kirkendall effect during the anion‐exchange reaction induced a slight hollowing within the core (Figure S4d,e, Supporting Information). To remove the buried nonfunctional Cu‐based species and provide better accessibility to the surface active sites, the core/shell CPS/*a‐*Ru_2_(P_0.9_Se_0.1_) NTs were treated with 3.0 m hydrochloric acid. The initial solid nanostructure with a CPS core gradually eroded into doubly tubular structured *a*‐Ru_2_(P_0.9_Se_0.1_) DWNTs (Figure S5, Supporting Information). After the catalysts were loaded onto Vulcan carbon (Vulcan XC‐72), subsequent treatments at 400 °C were carried out to enhance the structural robustness, resulting in the formation of *c‐*Ru_2_(P_0.9_Se_0.1_) DWNT/C (Figure 1b and S6a–d, Supporting Information).^[^ ^32^, ^33^ ^]^ By using the same protocol, *c‐*Ru_2_(P_0.8_Se_0.2_), *c‐*Ru_2_(P_0.7_Se_0.3_), and *c‐*Ru_2_(P_0.6_Se_0.4_) DWNTs were synthesized for catalytic performance comparison, and the ratio of Se was easily controlled by using different concentrations of the phenyl diselenide (PDSe) solution (Figure S6e, Supporting Information).

Previous investigations have reported that during electrochemical reactions, the amorphous and hydroxylated layers of atomic thickness spontaneously form on the surfaces of phosphide‐based catalysts.^[^ ^10^, ^34^ ^]^ Accordingly, it has been proposed that the presence of surface Ru–OH moieties is important for enhancing activity by facilitating the dissociation of water through chemical interaction with H_2_O molecules. To investigate the validity of this argument, we performed subsequent electrochemical processes to modify the surface structure of the *c*‐Ru_2_(P_0.9_Se_0.1_) DWNT/C and its associated catalytic properties employing cyclic voltammetry (CV) at 10 k cycles in a N_2_‐saturated 1.0 m KOH solution (+0.05–+0.20 V vs reversible hydrogen electrode [RHE]) at a sweep rate of 100 mV s^−1^.

After electrochemical treatment, Ru, P, and Se remained homogeneous in the *c*‐Ru_2_(P_0.9_Se_0.1_) DWNT/C, as revealed by energy‐dispersive X‐ray spectroscopy (EDS) mapping analysis (Figure S7, Supporting Information). X‐ray photoelectron spectroscopy (XPS) (Figure S8–S10, Supporting Information) and inductively coupled plasma atomic emission spectrometry analyses showed an Se content of ≈3.35 at %, in the *c*‐Ru_2_(P_0.9_Se_0.1_) DWNT/C (Figure S11 and S12, Supporting Information). In addition, the primary morphology and crystal structure of *c*‐Ru_2_(P_0.9_Se_0.1_) were fully maintained despite surface modification with the Ru–OH moieties (Figure 1c). The crystal structure of the activated *c‐*Ru_2_(P_0.9_Se_0.1_) DWNT/C was confirmed via the high‐angular annular dark‐field scanning transmission electron microscopy (HAADF‐STEM) imaging with sub‐ångström resolution and corresponding fast Fourier transforms (FFT) analysis, which revealed two different lattice fringes with interplanar distances of 0.232 and 0.194 nm corresponding to the (210) and (020) crystal planes of orthorhombic Ru_2_P, respectively (JCPDS No. 65‐2382) (Figure 1d). A comparison of the FFT pattern with the simulation results also supports the Ru_2_P crystal structure along the <001> direction (Figure 1e). The compositional and phasic evolution of the *c*‐Ru_2_(P_0.9_Se_0.1_) DWNT/C from CPS SWNTs were traced by the changes in the powder X‐ray diffraction (PXRD) patterns (Figure 1f). Elemental mapping and the corresponding line profiles of the activated catalysts revealed a reconstructed surface with Ru–OH species (Figure 1g,h and S8, Supporting Information), which is consistent with the XPS analysis (Figure 1i). The peak at around 461.7 eV corresponds to the characteristic Ru—P bond in Ru_2_P, while the peak at 464.0 eV is indicative of Ru–OH species.^[^ ^10^ ^]^ Notably, the Ru–OH peaks of the *c*‐Ru_2_(P_0.9_Se_0.1_) DWNT/C increased after the electrochemical process.

**Figure 2:**
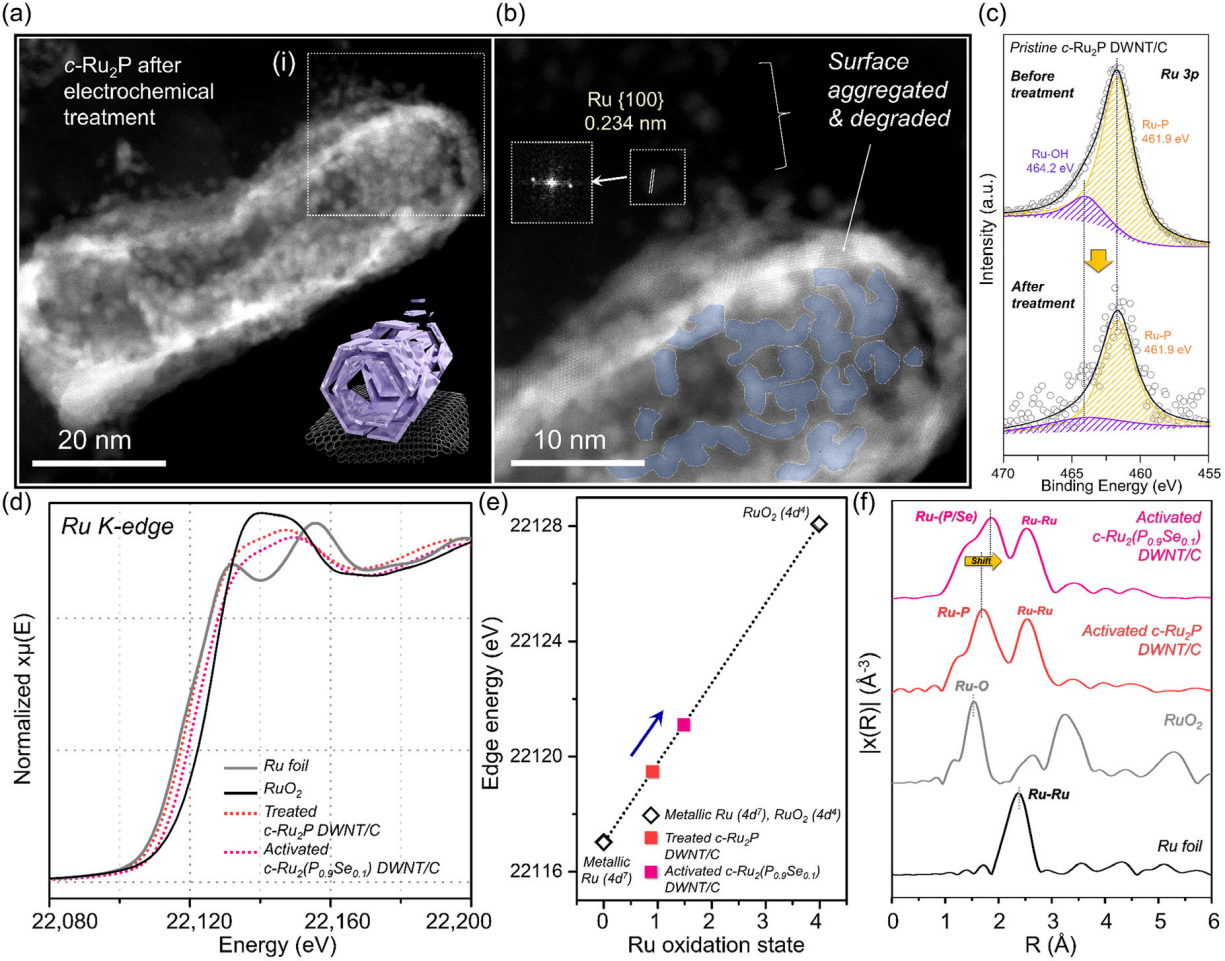
a,b) HADDF‐STEM image of *c*‐Ru_2_P DWNT after electrochemical treatment. c) Corresponding Ru 3*p* XPS of *c*‐Ru_2_P/C before and after electrochemical treatment. d) Ru K‐edge XANES profiles and e) the edge energies for Ru K‐edge as a function of the oxidation state of the Ru. f) FT‐EXAFS spectra of Ru K‐edge for treated *c*‐Ru_2_P DWNT/C and activated *c*‐Ru_2_(P_0.9_Se_0.1_) DWNT/C with Ru foil and RuO_2_ as references.

To elucidate the effect of Se doping on the catalytic performance of the Ru_2_P catalysts for the alkaline HER, we prepared pristine Ru_2_P phase catalysts (*c‐*Ru_2_P DWNT/C, Figure S13, Supporting Information), which were also electrochemically treated using the same procedure as *c*‐Ru_2_(P_0.9_Se_0.1_) DWNT/C. In contrast to the Ru–OH‐modified *c*‐Ru_2_(P_0.9_Se_0.1_) DWNT/C, which maintained its structural integrity, the morphology of the *c*‐Ru_2_P DWNTs was severely damaged forming multiple aggregates of small grains after the treatment (**Figure** **2**a). As revealed by high‐resolution STEM (HRSTEM) and EDS mapping analysis (Figure 2b and S14, Supporting Information), the as‐formed Ru aggregates were detached from the structure,^[^ ^35^ ^]^ implying the poor stability of the *c‐*Ru_2_P DWNT/C under alkaline HER. The structural degradation of *c*‐Ru_2_P DWNT/C was also observed in the XPS spectra (Figure 2c). Therefore, the augmented stability of the Ru_2_P phase through Se doping facilitates the retention of active Ru–OH moieties on the surface, mitigating structural degradation.

The effect of Se doping on the geometric and electronic structure engineering was further studied by scrutinizing the local coordination environment and chemical states via synchrotron‐based X‐ray absorption spectroscopy (XAS), employing a Ru metal foil (5*d* ^7^6*s* ^2^) and RuO_2_ (5*d* ^6^6*s* ^0^) powder as references. X‐ray absorption near‐edge structure (XANES) and extended X‐ray absorption fine structure (EXAFS) measurements provided a more detailed understanding of the valence states and coordination states of the Ru center in the activated *c*‐Ru_2_(P_0.9_Se_0.1_) and treated *c‐*Ru_2_P DWNT/C. As shown in Figure 2d, the XANES spectra of activated *c*‐Ru_2_(P_0.9_Se_0.1_) DWNT/C revealed that the white‐line adsorption energy of the Ru K‐edge was positioned between those of the Ru foil and RuO_2_ references and was more positive than that of the treated *c‐*Ru_2_P DWNT/C, indicating the presence of a Ru_2_P phase with Ru–OH‐modified surfaces (Figure 2e). The valence state results of the Ru species are consistent with those obtained from the XPS and HRSTEM analyses. In the FT‐EXAFS spectra, the activated *c*‐Ru_2_(P_0.9_Se_0.1_) and treated *c*‐Ru_2_P DWNT/C exhibit two prominent peaks at ≈2.5 and 1.7 Å, corresponding to the Ru—Ru and Ru—P scattering paths inherent to the characteristic Ru_2_P phase, respectively (Figure 2f and S15, Supporting Information).^[^ ^36^ ^]^ Notably, the first‐shell peak exhibited a discernible shift to higher R in the spectrum of the activated *c*‐Ru_2_(P_0.9_Se_0.1_) DWNT/C, indicative of an extended Ru—P bond length (2.27 Å) compared to that of the treated *c*‐Ru_2_P DWNT/C (2.24 Å) (Table S1, Supporting Information). The Ru—P bond length in the activated *c*‐Ru_2_(P_0.9_Se_0.1_) DWNT/C is slightly longer than that of the treated *c‐*Ru_2_P DWNT/C, suggesting the incorporation of larger Se anions into the pristine Ru_2_P phase.^[^ ^22^, ^27^ ^]^ As shown in Figure S16, Supporting Information, the simulated crystal models of the *c*‐Ru_2_(P_0.9_Se_0.1_) and pristine Ru_2_P coincide well with the experimental spectra, thereby confirming the geometrical changes induced by the incorporation of Se. The incorporation of Se alters the geometric and electronic structure, which strongly affects the catalytic properties of the Ru center in the Ru_2_P phase (vide infra).

### 2.2 Evaluation of the Electrochemical Hydrogen Evolution Performance

**Figure 3:**
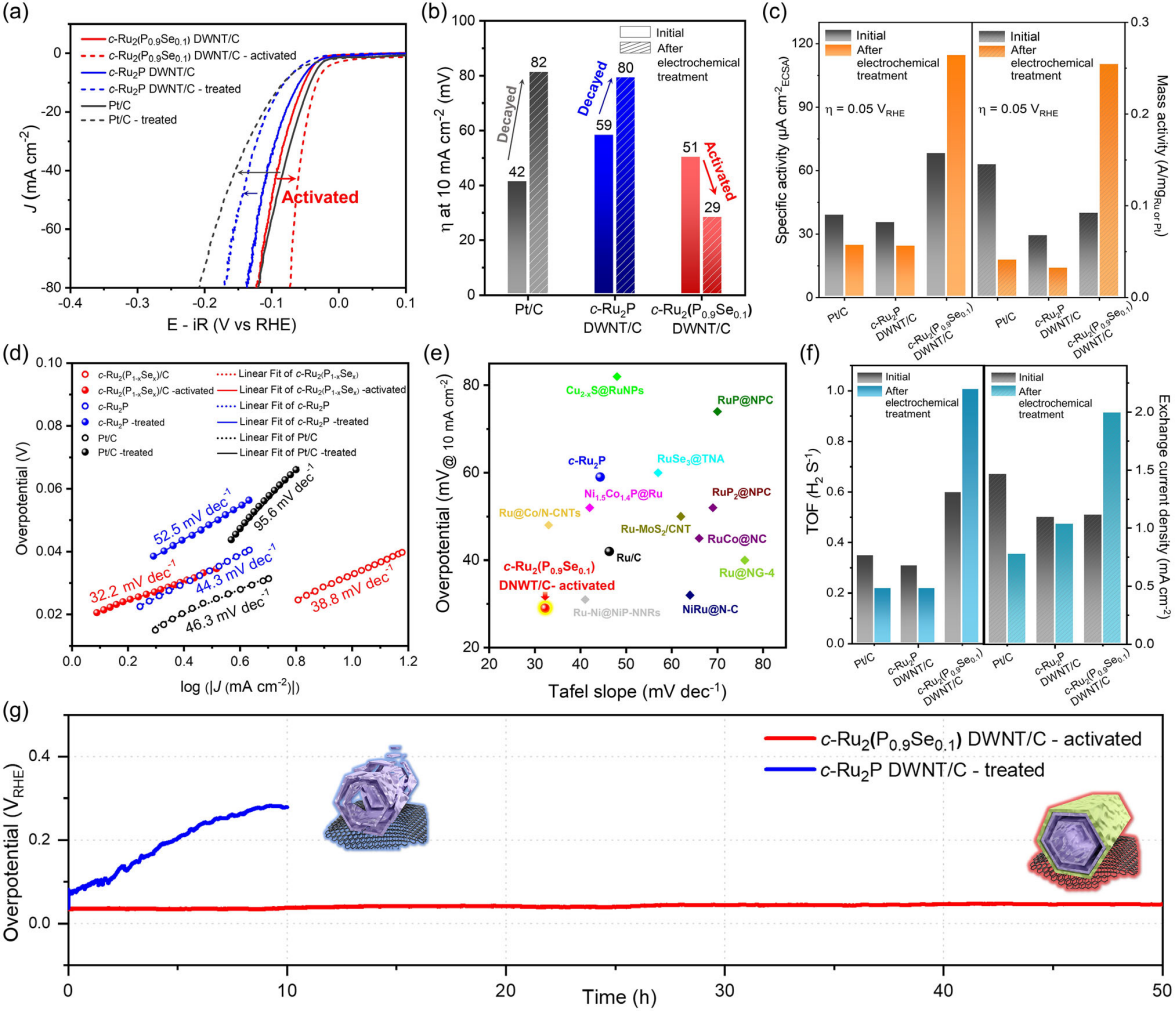
Electrocatalytic HER performance of *c‐*Ru_2_P DWNT/C, *c‐*Ru_2_(P_0.9_Se_0.1_) DWNT/C, Pt/C in N_2_‐saturated 1.0 m KOH. a) *iR*‐corrected HER polarization curves of the catalysts before and after electrochemical treatment. b) Overpotentials of catalysts derived from HER polarization curves at 10 mA cm^−2^. c) Specific and MA of the catalysts. d) Tafel slopes obtained from (a). e) The comparisons of Tafel slopes and overpotentials at 10 mA cm^−2^ with Ru‐based catalysts. f) TOFs and exchange current density of the catalysts. g) Chronopotentiometry response of electrochemically treated *c‐*Ru_2_P DWNT/C and *c‐*Ru_2_(P_0.9_Se_0.1_) DWNT/C at a constant cathodic current density of 10 mA cm^−2^.

The electrocatalytic HER performances of the *c‐*Ru_2_(P_0.9_Se_0.1_) DWNT/C, *c‐*Ru_2_P DWNT/C, and commercial Pt/C catalysts (Figure S17, Supporting Information) were evaluated in N_2_‐saturated 1.0 m KOH both before and after electrochemical treatment (**Figure** **3**a). The HER polarization curves revealed the enhanced HER activity of the *c‐*Ru_2_(P_0.9_Se_0.1_) DWNT/C, indicated by the reduced overpotential (*η*_10_) from 51 to 29 mV after the treatment. In contrast, the activities of the *c‐*Ru_2_P DWNT/C and Pt/C catalysts were severely reduced following electrochemical treatment (Figure 3b). The activated *c‐*Ru_2_(P_0.9_Se_0.1_) DWNT/C exhibited superior HER performance compared to the pristine and treated *c‐*Ru_2_P DWNT/C (*η*_10,pristine_ = 59 mV, *η*_10,treated_ 80 mV), as well as the commercial Pt/C (*η*_10,pristine_ = 42 mV, *η*_10,treated_ 82 mV). The optimal Se doping level in the *c*‐Ru_2_(P_0.9_Se_0.1_) DWNT/C was determined by analyzing the catalytic performance across various Se concentrations within the *c*‐Ru_2_(P_*x*_Se_*y*_) DWNT/C catalysts (detailed characterization presented in Figure S18 and S19, Supporting Information).

After electrochemical treatment, the Pt/C and *c*‐Ru_2_P DWNT/C catalysts showed a reduction in both electrochemical surface areas (ECSAs) and surface areas (SAs), which was attributed to the loss of their original morphologies due to dissolution. Conversely, the activated *c‐*Ru_2_(P_0.9_Se_0.1_) DWNT/C catalyst demonstrated an increase in ECSA and exhibited a much higher SA (114.9 μA cm_ECSA_^−2^) than the pristine *c‐*Ru_2_P DWNT/C (35.7 μA cm_ECSA_^−2^) and Pt/C (39.3 μA cm_ECSA_^−2^) at −50 mV (Figure 3c, S20, and S21, Supporting Information). The highly open and thin features of the double‐walled nanotube and robust crystal structure of activated *c‐*Ru_2_(P_0.9_Se_0.1_) DWNT/C may have enabled the maximized utilization of the active sites, while the *c‐*Ru_2_P DWNT/C loses its crystallinity during electrochemical treatment (Figure S22 and Note S3, Supporting Information). Furthermore, the mass activity (MA) and price activity (PA) of the catalysts were calculated, revealing that the activated *c*‐Ru_2_(P_0.9_Se_0.1_) DWNT/C catalyst exhibited ≈1.8 and 3.7 times higher MA and PA, respectively, than those of commercial Pt/C, indicating its cost‐effectiveness (Figure S23, Supporting Information). The superior HER activity of the *c‐*Ru_2_(P_0.9_Se_0.1_) DWNT/C was verified by its exceptionally smaller Tafel slope (32.2 mV dec^−1^) compared to those of the pristine *c‐*Ru_2_P DWNT/C (44.3 mV dec^−1^) and Pt/C (46.3 mV dec^−1^) (Figure 3d). In addition, the activated *c‐*Ru_2_(P_0.9_Se_0.1_) DWNTs exhibited a reduced charge‐transfer resistance (*R*_ct_) at 10 mV, which was as small as that of Pt/C (Figure S24 and Note S4, Supporting Information). These results indicate the facilitated HER kinetics of the Volmer–Tafel mechanism, assisted by the surface structural changes with the Ru–OH species due to Se doping. The alkaline HER performance of the activated *c‐*Ru_2_(P_0.9_Se_0.1_) DWNT/C was comparable or superior to that of state‐of‐the‐art Ru‐based catalysts (Figure 3e and Table S2, Supporting Information).

To quantitatively demonstrate the catalytic superiority of the activated *c‐*Ru_2_(P_0.9_Se_0.1_) DWNT/C, the turnover frequency (TOF), which represents the intrinsic electrocatalytic activity per active site, was calculated based on the estimated number of active sites using a well‐established method for phosphide materials reported by Jaramillo et al.^[^ ^37^, ^38^ ^]^ Figure 3f shows the TOF versus potential for the activated *c‐*Ru_2_(P_0.9_Se_0.1_) DWNT/C, *c‐*Ru_2_P, and Pt/C catalysts, where the TOF values followed the order activated *c‐*Ru_2_(P_0.9_Se_0.1_) DWNT/C > Pt/C ≥*c‐*Ru_2_P DWNT/C. The exchange current density (*j*_0_) is sensitive to the nature of the catalyst and determines the rate of the intrinsic electron transfer between the catalyst and the electrolyte solution.^[^ ^39^ ^]^ The *j*_0_ value of the activated *c‐*Ru_2_(P_0.9_Se_0.1_) DWNTs was 2.01 mA cm^−2^, which is approximately eightfold and twofold higher than those of the *c‐*Ru_2_P DWNTs/C (1.10 mA cm^−2^) and Pt/C (1.47 mA cm^−2^), respectively. These results imply that the activated *c‐*Ru_2_(P_0.9_Se_0.1_) DWNT/C exhibits a remarkably high intrinsic activity, emphasizing the importance of the well‐defined active Ru–OH layers stabilized by the *c‐*Ru_2_(P_0.9_Se_0.1_) substrates.

To assess the durability of the catalyst, which is another important criterion for electrocatalyst evaluation, we conducted accelerated durability test and a long‐term durability test via time‐dependent chronopotentiometric measurements. After continuous 15 K cycling in 1.0 m KOH, the polarization curve of the activated *c‐*Ru_2_(P_0.9_Se_0.1_) DWNT/C presented only slight negative shift of 4.8 mV at a current density of 10 mA cm^−2^, which is superior to that of the *c‐*Ru_2_P DWNT/C and Pt/C (Figure S25, Supporting Information). Moreover, within 50 h long‐term operation at a constant current density of 10 mV cm^−2^, negligible current fluctuations were observed, highlighting the robustness of the *c‐*Ru_2_(P_0.9_Se_0.1_) phase (Figure 3g).

### 2.3 AEMWE Sing‐Cell Performance of *c*‐Ru_2_(P_0.9_Se_0.1_) DWNT/C

**Figure 4:**
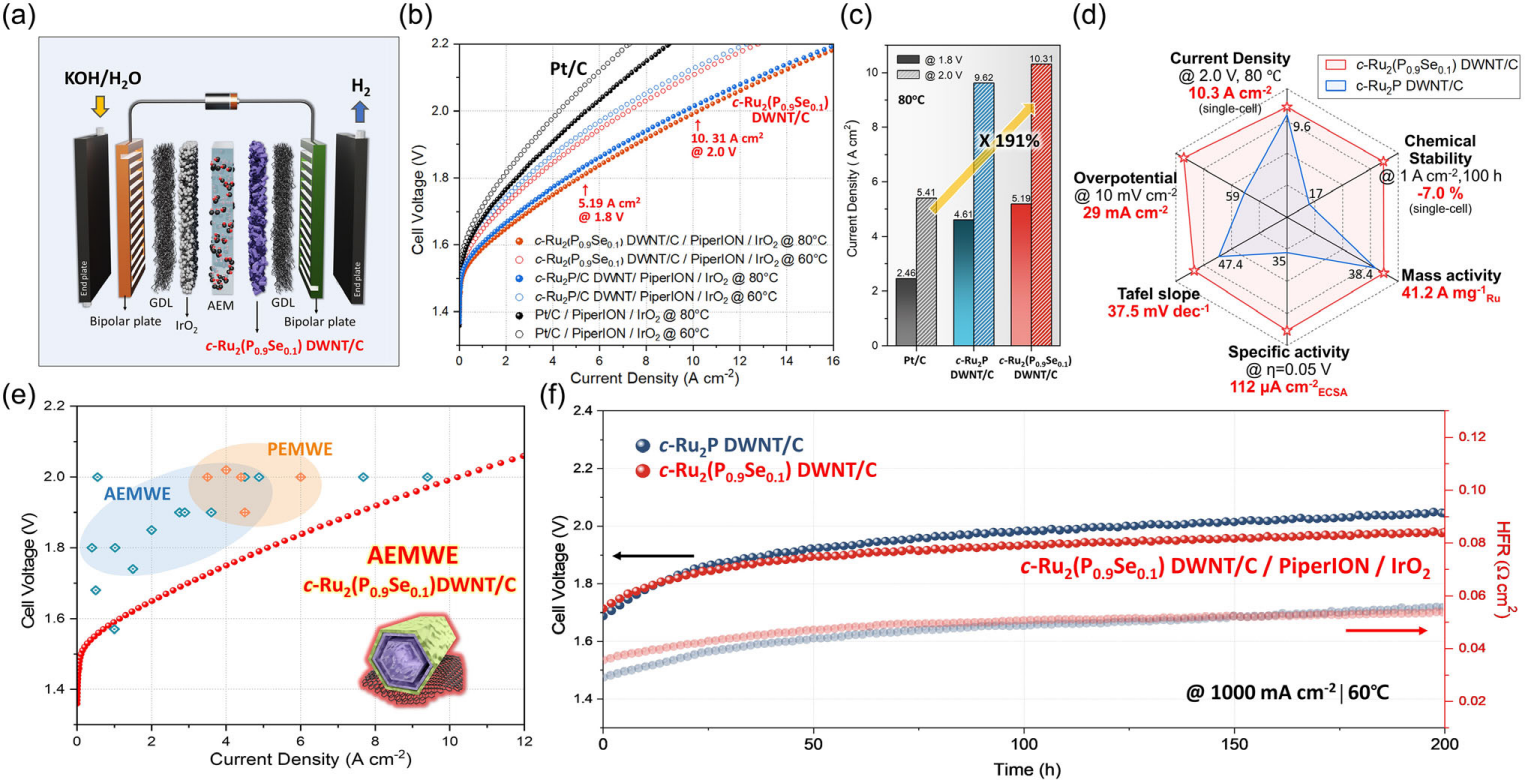
Single‐cell test for the AEMWE. a) Schematic illustration of AEMWE device. b) Polarization curves (*I*–*V* curves) of AEMWEs using Pt/C, *c‐*Ru_2_P DWNT/C, and *c‐*Ru_2_(P_0.9_Se_0.1_) DWNT/C at 60 and 80 °C. c) Comparison of performances (current densities at 1.8 and 2.0 V). d) Normalized radar chart comparing the performances including stability of the *c‐*Ru_2_P DWNT/C and activated *c‐*Ru_2_(P_0.9_Se_0.1_) DWNT/C. e) Comparison of reported AEMWEs and PEMWEs with the advanced AEMWE using the *c‐*Ru_2_(P_0.9_Se_0.1_) DWNT/C. f) Durability cell voltage–time plots for the AEMWE based on *c‐*Ru_2_(P_0.9_Se_0.1_) DWNT/C at a constant current density of 1000 mA cm^−2^ at 60 °C. All measurements were performed after the activation process of *c‐*Ru_2_(P_0.9_Se_0.1_) DWNT/C.

Although research on alkaline HER electrocatalysts has gained significant attention, studies on the evaluation of catalysts implemented in practical AEMWEs for industrial applications are scarce.^[^ ^40^, ^41^ ^]^ Encouraged by the excellent HER activity, we constructed a membrane electrode assembly (MEA) in an AEMWE by using *c‐*Ru_2_(P_0.9_Se_0.1_) DWNT/C as the cathode to evaluate its performance in a practical water electrolyzer (**Figure** **4**a and S26, Supporting Information). In this study, MEAs were fabricated from *c‐*Ru_2_(P_0.9_Se_0.1_) DWNT/C, *c‐*Ru_2_P DWNT/C, and commercial Pt/C (20 wt%) (2.0 mg cm^−2^) with 0.25 mg_Ru(Pt)_ cm^−2^ and were compared in an AEMWE system. Each electrolyzer was then tested in a flowing 1.0 m KOH solution at different temperatures (60 and 80 °C). The *c‐*Ru_2_(P_0.9_Se_0.1_) DWNT/C catalyst was evaluated after the break‐in procedure, which activated the catalyst layer. As shown in Figure 4b, the MEA performance of the activated *c‐*Ru_2_(P_0.9_Se_0.1_) DWNT/C can be further enhanced by adopting the break‐in procedure, exhibiting low onset‐cell voltages and an outstanding current density of 5.19 A cm^−2^ at 1.8 V and 80 °C. The current density of the electrolyzer rose to 10.31 A cm^−2^ at 2.0 V, along with a decrease in the ohmic and charge transfer resistances (Figure S27, Supporting Information). In addition, the MA value of MEA (activated *c‐*Ru_2_(P_0.9_Se_0.1_) DWNT/C) reached 41.2 A mg_Ru_^−1^ at 2.0 V, representing an activity of more than 1.9‐fold higher compared with that of the com. Pt/C (Figure 4c). The efficient water penetration and extensive SA of the *c‐*Ru_2_(P_0.9_Se_0.1_) DWNT/C compared to the *c‐*Ru_2_P DWNT/C were validated through molecular dynamics (MD) simulations, substantiating its exceptional performance in AEMWE (Figure S33 and S34, Supporting Information). Based on its remarkable activity, the durability of the electrolyzer was evaluated at a constant current of 1.0 A cm^−2^ in 1.0 m KOH at 60 °C (Figure 4f and S28, Supporting Information). The durability of the activated *c*‐Ru_2_(P_0.9_Se_0.1_) DWNT/C was demonstrated over 200 h with a slight increased rate of 1.1 mV h^−1^, revealing its excellent structural robustness compared to the *c*‐Ru_2_P DWNT/C (1.5 mV h^−1^) and com. Pt/C (1.8 mV h^−1^). To the best of our knowledge, this cell performance is among the best reported competitive alkaline, PEM (proton‐exchange membrane), and AEM electrolytic performances (Figure 4d,e and Table S3, Supporting Information).

### 2.4 DFT Calculations

**Figure 5:**
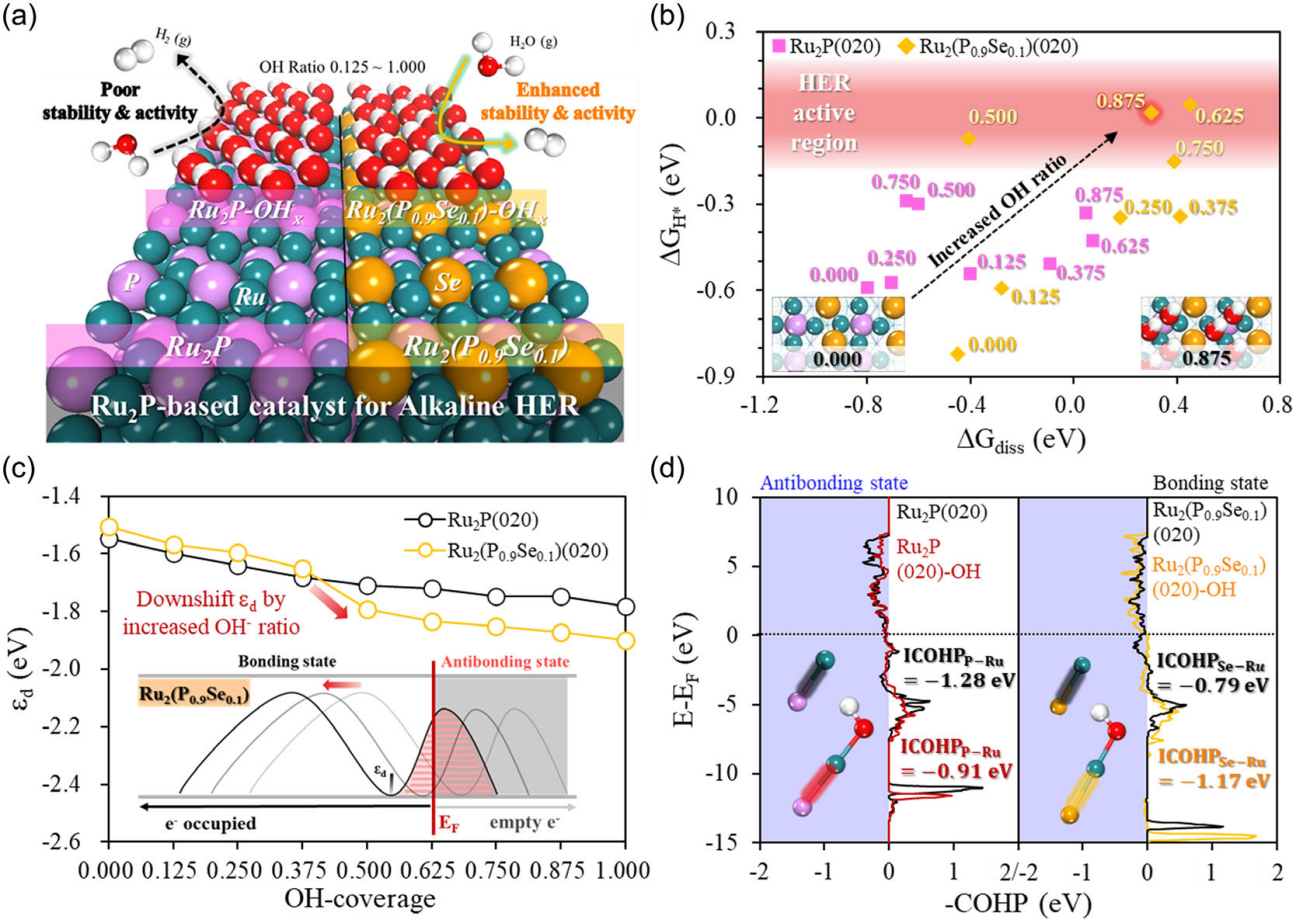
a) Schematic structures of Ru−OH‐modified Ru_2_P‐based catalysts. b) Correlation plot between $\Delta G_{H^{*}}$ and $\Delta G_{\text{diss}}$ over the OH* coverage, where the red shaded area represents the effective HER active region (|$\Delta G_{H^{*}}$| ≤ 0.2 eV). c) Estimated *d*‐band center value (*ε*_d_) of the Ru_2_P(020) and Ru_2_(P_0.9_Se_0.1_)(020) catalysts depending on OH* coverage with a schematic illustration of antibonding state occupation in the PDOS. d) Separated orbital states of the P—Ru and Se—Ru bond, respectively, by COHP analysis including integrated COHP (ICOHP) values below the Fermi level.

Density functional theory (DFT) calculations were performed to gain insights into the origin of the enhanced alkaline HER performance and stability of the *c*‐Ru_2_(P_0.9_Se_0.1_) DWNT/C catalyst compared with *c*‐Ru_2_P DWNT/C through Se doping. Based on the experimental observations as shown in Figure 1, we constructed the pristine and Se‐doped catalysts, Ru_2_P(020) and Ru_2_(P_0.9_Se_0.1_)(020), respectively, as Ru_2_P‐based catalysts. Thermodynamic evaluation was conducted by estimating the Se substitution energy ($E_{\text{Sub}}^{\text{Se}}$) according to the surface structure (Figure S29, Supporting Information). To investigate the feasibility of the experimentally observed Ru–OH‐modified catalysts at the electrochemical activation process (Figure 1b,c), we systematically designed the OH*‐covered Ru_2_P‐based catalysts, Ru_2_P(020)–OH_*x*_ and Ru_2_(P_0.9_Se_0.1_)(020)–OH_*x*_ (0.125 ≤ *x* ≤ 1.000), as shown in **Figure** **5**a. The integral adsorption free energy of OH* ($\Delta G_{{\text{OH}}^{*}}^{\text{Int}}$) was then investigated according to the OH coverage, which confirmed that the continuous binding of OH* to the catalyst surface up to a complete 100% coverage is thermodynamically favorable for both the Ru_2_P and Ru_2_(P_0.9_Se_0.1_) catalysts (Figure S30, Supporting Information). Therefore, the Ru–OH‐modified Ru_2_P and Ru_2_(P_0.9_Se_0.1_) catalysts can be produced through an electrochemical activation process and are expected to serve as practical catalyst for HER in an alkaline environment.

Given the well‐established Ru–OH‐modified Ru_2_P and Ru_2_(P_0.9_Se_0.1_) catalysts, we constructed free energy diagrams (FED) depending on the degree of OH* coverage, considering the intermediates of H_2_O* (adsorption), H*/OH* (dissociation), and H* (H_2_ production) in the alkaline HER process (Figure S31, Supporting Information).^[^ ^42^, ^43^, ^44^ ^]^ As shown in Figure S32, Supporting Information, the calculated FED demonstrates that the $\Delta G_{H_{2}O^{*}}$ values are sufficient to initiate alkaline HER via binding of H_2_O ($\Delta G_{H_{2}O^{*}}\leq 0.1\text{ eV}$) on the catalyst surface although the H_2_O binding energies ($\Delta G_{H_{2}O^{*}}$) are slightly weakened by increasing the OH* coverage in both the Ru_2_P(020) and Ru_2_(P_0.9_Se_0.1_)(020) catalysts. Interestingly, at high OH* coverage (*x* ≥0.5), the calculated hydrogen binding energies ($\Delta G_{H^{*}}$) at the Ru sites of each catalyst as HER descriptors show that the Ru_2_(P_0.9_Se_0.1_)(020) catalysts have an appropriate $\Delta G_{H^{*}}$ value of |$\Delta G_{H^{*}}$| ≤ 0.2 eV^[^ ^17^ ^]^ resulting in a higher HER activity compared to Ru_2_P(020), which has a much more negative $\Delta G_{H^{*}}$ value. However, for the Ru_2_(P_0.9_Se_0.1_)(020) catalysts with moderate OH* coverage, the dissociation of H_2_O* into H* and OH* is slightly endothermic, which corresponds to the energy barrier that must be overcome for the reaction to proceed. Therefore, the H_2_O* dissociation and H_2_ production reactions are crucial factors in determining the HER activity of the Ru_2_P‐based catalysts. To further clarify the effect of Se doping and OH* coverage on $\Delta G_{\text{diss}}$ ($\Delta G_{H^{*}/{\text{OH}}^{*}}-\Delta G_{H_{2}O^{*}}$) and $\Delta G_{H^{*}}$, the correlation plot was analyzed as shown in Figure 5b. The correlation plot shows that the $\Delta G_{H^{*}}$ values of both the Ru_2_P(020) and Ru_2_(P_0.9_Se_0.1_)(020) catalysts shifted upward, toward the HER‐active region, with increasing the OH* ratio. While the Ru_2_P(020) catalysts still failed to reach the HER‐active region (|$\Delta G_{H^{*}}$| ≤ 0.2 eV), the Ru_2_(P_0.9_Se_0.1_)(020) catalysts clearly achieved it in the high OH* coverage (*x* ≥0.5). Notably, Ru_2_(P_0.9_Se_0.1_)(020)–OH_0.875_ showed a superior HER activity despite a slightly positive $\Delta G_{\text{diss}}$ value, which can be sufficiently overcome under the HER operating conditions. Based on these results, we confirmed that the Ru–OH‐modified Ru_2_(P_0.9_Se_0.1_)(020) is a superior HER catalyst to Ru_2_P(020) through Se doping and OH* coverage surface modification.

To reveal the enhanced catalytic activity of the Ru–OH‐modified Ru_2_(P_0.9_Se_0.1_)(020), we conducted a partial density of state (PDOS) analysis depending on the degree of OH*‐coverage of the Ru_2_P‐based catalyst. The bond strength of an adsorbate on the catalyst surface is closely related to the filling of the antibonding state near the Fermi level, which is estimated by the *d*‐band center (*ε*_d_) theory of Hammer and Norskov.^[^ ^45^ ^]^ The increased filling of the antibonding state led to the weak binding energy of the adsorbates. As shown in Figure 5c, the *ε*_d_ values of the Ru_2_P(020) and Ru_2_(P_0.9_Se_0.1_)(020) surfaces shift downward with increasing OH* coverage, which implies a weaker binding strength between the adsorbate and the catalysts. Interestingly, when OH* covers more than half of the surface (*x* ≥0.5), the *ε*_d_ values of Ru_2_(P_0.9_Se_0.1_)(020) dramatically shift downward than those of Ru_2_P(020). This unique tendency indicates that the electronic structure of the Se‐doped surface is more sensitive to the surface modification of the OH* coverage which leads to the enhanced HER activity of the Ru sites near Se through the optimized electric structures.

To explore the origin of the enhanced durability of Ru_2_(P_0.9_Se_0.1_)(020) compared to Ru_2_P(020), the chemical bonds of P—Ru and Se—Ru within the Ru–OH‐modified surface structures were analyzed via the crystal orbital Hamilton population (COHP). The distributions of the bonding and antibonding states of the P—Ru and Se—Ru bond in the Ru–OH‐modified surface structures are shown in Figure 5d. The analysis showed that Ru–OH modification afforded opposite effects on the P—Ru and Se—Ru bonds. That is, Ru–OH modification increased the Se—Ru bonding state of Ru_2_(P_0.9_Se_0.1_)(020), while it decreased the P—Ru bonding state of Ru_2_P(020). Moreover, integrating the COHP value up to the Fermi level (ICOHP) clearly indicates that Ru–OH modification promotes a stronger Se—Ru bond with a negatively decreased ICOHP value from −0.79 to −1.17 eV compared to weakening the P—Ru bonding with an increasing ICOHP value from −1.28 to −0.91 eV. These results reveal that the Ru–OH‐modified Ru_2_(P_0.9_Se_0.1_)(020) has a higher cohesive energy than Ru_2_P(020)–OH owing to the strong interaction between Se and Ru, effectively preventing structural degradation.

## 3 Conclusion

In this study, we developed an Se‐doped Ru_2_P double‐walled nanotube catalyst, which demonstrated outstanding catalytic activity and durability toward the alkaline HER after electrochemical priming, with a low overpotential of 29 mV at 10 mA cm^−2^. Experimental and theoretical calculation results revealed that the surface‐doped Se enhanced the structural stability of the active Ru–OH species on the catalyst surface by considerably modifying the electronic structures around the catalytically active sites. Furthermore, the AEMWE device incorporating the *c‐*Ru_2_(P_0.9_Se_0.1_) DWNT/C showed a promising performance of 10.31 A cm^−2^ at 80 °C and a stable cell voltage over 200 h at a constant current density of 1000 mA^−2^, single‐handedly exceeding the single‐cell activity of commercial Pt/C. The electronic structure engineering of Ru_2_P induced by the Se dopant, along with the achievement of an extremely thin, high SA, 2D structural motif from the conversion chemistry of solid nanoparticles, could open broad avenues for the development of promising non‐Pt‐based electrocatalysts for practical AEMWEs applications.

## 4 Experimental Section

4.1

4.1.1

##### Chemicals

Copper (II) nitrate trihydrate [Cu(NO_3_)_2_·3H_2_O, puriss. p.a. 99–104%], oleylamine (70%, OAm), 1‐octadecene (90%, ODE), trioctylphosphine oxide [TOPO, ≥90%], *tert*‐dodecanethiol [*t‐*DDT, mixture of isomers 98.5%], 1‐dodecanethiol [1‐DDT, ≥98%], tris(diethylamino)phosphine (97%, TDP), Ru (III) acetylacetonate [Ru(acac)_3_, 97%], and PDSe (98%) were purchased from Sigma–Aldrich. Hydrochloric acid (HCl, 35%) was purchased from Daejung Chemicals & Metals Co. Ltd., Korea. Carbon black (Vulcan XC‐72) was purchased from Cabot Corporation, USA. Pt/C (platinum 20% on the carbon black) was purchased from Alfa Aesar. All chemicals were used as received without further purification.

##### Synthesis of CPS NTs

The synthesis of the CPS SWNTs involved the synthesis of hexagonal roxbyite CS NRs following a previously reported method.^[^ ^29^ ^]^ The prepared CS NRs were dispersed in 7 mL OAm and the solution was placed in a 100 mL Schlenk tube. The Schlenk tube was vacuumed at 100 °C for 10 min and then charged with Ar (1 atm) after being cycled twice with Ar and vacuum. The tube was placed into a preheated oil bath at 240 °C and 0.750 mL TDP filled with Ar was immediately injected into the solution. The reaction mixture was kept at 240 °C for 30 min and the degree of phosphorization was modulated by controlling the reaction time. The mixture was then cooled to room temperature under magnetic stirring. The blackish precipitate was washed with 20 mL of toluene and ethanol, followed by centrifugation at 4000 rpm for 5 min. This process was conducted in triplicate, and the precipitated CPS SWNTs was dried under vacuum for further cation‐exchange reactions.

##### Cation‐Exchange of CPS SWNTs with Ru for Cu_*3–x*_ *P–S/amorphous Ru*_*2*_ *P Nanotubes (CPS/RP NTs)*

The previously synthesized CPS SWNTs powder (20 mg) was dissolved in 5 mL of OAm. A slurry containing 0.1 mmol of Ru(acac)_3_ and the CPS SWNTs solution was prepared in a 100 mL Schlenk tube with a magnetic stirrer. The tube was placed at 100 ° Cunder vacuum for 10 min. After charging the tube with Ar (1 atm), the tube was directly transferred into a preheated oil bath at 200 °C and kept at this temperature for 1 h upon vigorous stirring. After the reaction, the mixture was cooled to room temperature and washed 3 times with toluene and ethanol. The washing procedure described above was followed.

##### Synthesis of Se‐Doped CPS/Amorphous Ru_*2*_ *P Core/Shell (CPS/a‐Ru*_*2*_ *(P*_*0.9*_ *Se*_*0.1*_ *) Core/Shell NTs)*

To prepare the CPS/*a‐*Ru_2_(P_0.9_Se_0.1_) core/shell NTs, 10 mg of the CPS/*a‐*Ru_2_P core/shell NTs powder was dissolved in 5 mL of OAm. After transferring the solution into a 100 mL Schlenk tube, the tube was held under vacuum at 100 °C for 10 min and then charged with Ar (1 atm). The Schlenk tube was directly placed into a preheated oil bath at 200 °C. A PDSe–OAm solution (0.01 m) was prepared by dissolving 0.031 g of PDSe in 10 mL of OAm and was rapidly injected into the solution. The reaction mixture was kept at 200 °C for 1 h. The same cooling and washing steps described for the CPS/*a‐*Ru_2_P NTs were followed.

##### Chemical Etching Process for a‐Ru_*2*_ *P DWNTs and a‐Ru*_*2*_ *(P*_*0.9*_ *Se*_*0.1*_ *) DWNTs*

To remove the CPS core and afford *a‐*Ru_2_P DWNTs and *a‐*Ru_2_(P_0.9_Se_0.1_) DWNTs, a chemical etching process was performed. The resulting dried powder (8 mg) was dispersed in 2 mL of toluene and 2 mL of ethanol and then mixed with 2 mL of a 3.0 m HCl solution. The mixture was held at 60 °C for 1 h. After the precipitated *a‐*Ru_2_P (or *a‐*Ru_2_(P_0.9_Se_0.1_) DWNTs) was cooled to room temperature, 25 mL of ethanol was added, followed by centrifugation at 3500 rpm for 5 min. This washing procedure was repeated using ethanol. The resulting precipitate was redispersed in OAm (2 mL) and the mixture was collected via centrifugation at 4000 rpm for 5 min after washing with 10 mL of toluene and 20 mL of ethanol.

##### Thermal Annealing of Carbon Supported Catalysts

The prepared *a‐*Ru_2_P DWNTs and *a‐*Ru_2_(P_0.9_Se_0.1_) DWNTs (10 mg) were dispersed in chloroform (5 mL). Carbon black (Vulcan XC‐72, 10 mg) was dispersed in chloroform (20 mL) and sonicated for 15 min. A suspension of Se‐doped Ru_2_P NTs (or Ru_2_P NTs) in 5 mL of chloroform was added dropwise to the carbon black‐containing slurry, and the mixture was further sonicated for 15 min in an ice bath. The *a‐*Ru_2_P DWNT/C and and *a‐*Ru_2_(P_0.9_Se_0.1_) DWNT/C catalysts were separated by repeated washing with ethanol, and the obtained products were dried under vacuum. The catalysts were further annealed at 400 °C in a 5% H_2_/Ar atmosphere for 10 min.

##### Characterization

TEM and HRTEM analyses were performed using a TECNAI G2 20 S‐twin instrument operated at 200 kV and a TECNAI G2 F30ST instrument operated at 300 kV, respectively. Aberration‐corrected imaging and high spatial resolution EDS analyses were performed at the FEI Nanoport in Eindhoven using a Titan Probe Cs TEM 300 kV with Chemi‐STEM technology. PXRD patterns were collected with a Rigaku Ultima III diffractometer system using Cu Kα radiation at 40 kV and 30 mA. XPS analysis was conducted using a ULVAC‐PHI X‐tool X‐ray photoelectron spectrometer equipped with a monochromatic Al Kα radiation source (1486.6 eV) operating at 24.1 W. The C 1*s* line at 284.5 eV was used as the reference line. XAS including XANES and EXAFS analyses of the Ru K‐edge were conducted using beamlines 7D, 8C, and 1C at the Pohang Accelerator Laboratory (PAL) in the Republic of Korea. The XAS measurements were performed in fluorescence‐transmission geometry, where the spectra of the samples and standards were measured in the fluorescence mode, and the spectra of the reference materials, such as Ru foil placed behind the datasets, were obtained at room temperature. The Ru K‐edge XANES spectra were calibrated at 22 117 eV.

##### Electrochemical Characterization

Electrochemical characterization was conducted in N_2_‐saturated 1.0 m KOH (ACS reagent, ≥85%, Merck) using a CHI 7007 E electrochemical analyzer (CH Instrument, Inc.) at room temperature. A conventional three‐electrode system consisting of a rotating disk electrode (RDE, 0.1963 cm^−2^) as the working electrode, a graphite rod as the counter electrode, and Hg/HgO (1.0 m NaOH) as the reference electrode was used. Calibration was performed using a sealed standard three‐electrode system with a Pt mesh serving as both the counter and working electrodes. The electrolyte (1.0 m KOH) was saturated with high‐purity H_2_ for a minimum of 30 min before calibration. CV cycles with two sweep segments were obtained at a scan rate of 1 mV s^−1^. This value was experimentally measured following a calibration process described in the literature.^[^ ^46^, ^47^ ^]^ All potentials in this study were *iR*‐corrected to remove the ohmic drop across the electrolyte and referenced to a reversible hydrogen electrode.

*E*_RHE_ = *E*_Hg/HgO_ + *E* ^o^_Hg/HgO_ + 0.059 × pH = *E*_Hg/HgO_ + 0.925 V (the theoretical value is 0.924 V at 1.0 m KOH)

To prepare the working electrode, ≈3.0 mg of each catalyst (50% loaded on Vulcan carbon) was mixed with 150 μL of isopropyl alcohol, 350 μL of deionized water, and 15 μL of Nafion (5 wt%, Alfar Aesar), followed by sonication for 30 min in an ice bath. RDE was polished with a 1 and 0.05 μm alumina suspension on a polishing pad (Buehler). After that, the as‐prepared ink (9 μL) was dropped on RDE. The amount of noble metal on the GC electrode was 20 μg_Ru_ cm^−2^ for all catalysts. All data were obtained after conversion to the RHE scale by measuring the open‐circuit potential of Ag/AgCl with RHE (H^+^|H_2_ equilibrium on the Pt mesh). Before evaluating the HER activity, electrochemical cleaning was performed via CV cycles in a potential range of –0.3 to 0.15 V_RHE_ at a scan rate of 50 mV s^−1^ (five cycles). Thereafter, linear sweep voltamemetry (LSV) was conducted to measure the HER activity in a potential range of –0.5–0.2 V_RHE_ at a scan rate of 10 mV s^−1^ under 1600 rpm of electrode rotation. Electrochemical impedance spectroscopy (EIS) was conducted from 10 000 to 1 Hz at 0.05 V_RHE_ with an amplitude of 10 mV. Long‐term cycle testing was carried out for 15 000 CV cycles over the –0.05 to –0.2 V_RHE_ potential range at a scan rate of 100 mV s^−1^.

##### ECSA Measurements

The electrochemically active surface area (ECSA) was estimated from the double‐layer capacitance. The double‐layer capacitance (*C*_dl_) was assessed via differential capacitance measurements at different scan rates in the non‐Faradaic region. CVs were measured in the potential window of 0.5–0.7 V_RHE_ at scan rates of 10, 20, 40, 60, 80, and 100 mV s^−1^. *C*_dl_ is expressed by Equation (1).

(1) $$i_{c}=vC_{\text{dl}}$$

where $i_{c}$ is the measured charging current and *v* is the scan rate.

ECSA is derived from dividing the $C_{\text{dl}}$ with the specific capacitance $C_{s}$ (Equation (2)).

(2) $$\text{ECSA}=C_{\text{dl}}/C_{s}$$

In this study, the specific capacitance $C_{s}=0.0040{\text{ mF cm}}^{-2}$ was used to calculate ECSA based on typical reported values (Equation (3)).

(3) $$\text{TOF}=\frac{\#\text{total hydrogen turn overs}/{\text{cm}}^{2}\text{geometric area}}{\#\text{active sites}/{\text{cm}}^{2}\text{geometric area}}$$

The total number of H_2_ turnovers was calculated from the current density, as follows:

# of H_2_ = $\left(j\frac{\text{mA}}{{\text{cm}}^{2}}\right)\;\left(\frac{1{\text{Cs}}^{-1}}{1000\text{ mA}}\right)\;\left(\frac{1{\text{mol e}}^{-}}{96485.3\text{ C}}\right)\;\left(\frac{1{\text{ mol H}}_{2}}{2{\text{ mol e}}^{-}}\right)\;\left(\frac{6.022\times 1023{\text{ H}}_{2}\text{molecules}}{1{\text{ mol H}}_{2}}\right)$

= 3.12 × 10^15^ $\frac{H_{2}/s}{{\text{cm}}^{2}}\text{per}\frac{\text{mA}}{{\text{cm}}^{2}}$

The active sites per unit real SA were calculated using Equation (4).

(4) $$\text{Active sites}=\left(\frac{\text{ atoms}/\text{unit cell}}{/\text{unit cell}}\right)={\text{atoms cm}}_{\text{real}}^{-2}$$

The current density plot was converted into a TOF plot using Equation (5).

(5) $$\text{TOF}=\frac{\left(3.12\times 1015\;\frac{\frac{H_{2}}{s}}{{\text{cm}}^{2}}\text{per}\frac{\text{mA}}{{\text{cm}}^{2}}\right)\times \left|j\right|}{{\text{atoms cm}}_{\text{real}}^{-2}\times A_{\text{ECSA}}}$$

where *j* denotes the current density at 100 mV, *A* denotes the SA of the GC electrode, and *F* is the Faraday constant (96485.3 C mol^−1^).

##### Single‐Cell (Anion‐Exchange Membrane Water Electrolyzer) Test

All the MEAs were prepared using the catalyst‐coating method. Commercial IrO_2_ (99.99% Alfa Aesar) was utilized as the anode, while Pt/C (Alfa Aesar) and the synthesized *c‐*Ru_2_(P_0.9_Se_0.1_) DWNTs served as catalysts for the cathode. The AEM utilized was a PiperION membrane with a thickness of 20 μm. For both the anode and the cathode, a 5 wt% PiperION anion‐exchange dispersion served as the ionomer. The catalyst‐to‐ionomer weight ratios were set to 10 and 30 wt% for the anode and cathode, respectively. The catalyst loading was 2.0 mg_Ir_ cm^−2^ for the anode and 0.25 mg_Ru(Pt)_ cm^−2^ for the cathode, applied using a spray machine. In the AEMWE test, MEAs were compiled with single‐cell components, including end plates, bipolar plates, gas diffusion layers (GDLs), and gaskets. The anode‐side GDL was made of stainless steel fiber paper (68 841, Dioxide Materials), while nickel fiber paper (68 844, Dioxide Materials) was used on the cathode side. GDLs were pretreated with 30 wt% NaOH (98%, DAEJUNG) for 30 min at 90 °C, followed by treatment with 20 wt% HCl (DAEJUNG) in a sonication bath for 10 min for the cathode GDL. After each treatment, GDLs were rinsed with deionized water to remove any impurities. Before the assembly of the single cell, the membranes coated with the catalysts were pretreated with 6.0 m KOH and 1.0 m KOH for one hour each. Single‐cell testing was performed using 1.0 m KOH (90%, Merck) at ambient pressure, delivering 1.0 m KOH to both the anode and cathode sides at a rate of 30 mL min^−1^. The active area of the cell was 5 cm^2^, and the cell temperatures were maintained at 60 or 80 °C. Electrochemical measurements were performed with an HCP‐803 (Bio‐Logic, France), where LSV ranged from 1.35 to 2.2 V at a scan rate of 10 mV s^−1^. EIS tests were performed at 1.6, 1.8, and 2.0 V across frequencies from 50 kHz to 50 MHz with an amplitude potential of 10 mV. Durability testing was conducted using the constant current method at 5.0 A (1.0 A cm^−2^) for 200 h.

##### Computational Detail

All ab initio calculations were performed using the Vienna ab initio simulation package (VASP 5.4.4).^[^ ^48^, ^49^ ^]^ The projector augmented wave (PAW) method^[^ ^50^, ^51^ ^]^ was employed, and the exchange–correlation interactions were treated through the Perdew–Burke–Ernzerohf^[^ ^52^ ^]^ functional under the generalized gradient approximation. Integration in the Brillouin zone was performed based on the Monkhorst–Pack scheme using 3 × 4 × 6 and 2 × 2 × 1 *k*‐point meshes in each primitive lattice vector of the reciprocal space for geometry optimization of the Ru_2_P bulk and Ru_2_P(020) surface structures, respectively.^[^ ^53^ ^]^ The DFT‐D3 dispersion correction method was used to reflect the nonbonding interactions correlation in the Ru_2_P‐based catalyst.^[^ ^52^, ^54^ ^]^ The lattice constants and internal atomic positions were fully optimized using a plane‐wave cutoff energy of 500 eV and spin‐polarized calculations until the residual forces were less than 0.04 eV Å^−1^. Detailed information on the catalytic activity evaluation for the alkaline HER is provided in Note S1, Supporting Information. COHP analysis was employed to reveal the chemical bonds by dividing the electronic structure into bonding and antibonding contributions using the local orbital basis suite toward the electronic structure reconstruction (LOBSTER) code based on the pbeVaspFit2015 basis set.^[^ ^43^, ^55^ ^]^ Classical MD simulations were systemically performed using the FORCITE module in the Material Studio 2022 (BIOVIA, San Diego, CA) with universal force fields^[^ ^56^ ^]^ to simulate the dynamics of water on the Ru_2_P and Se‐doped Ru_2_P surface structures. Slab models for the MD simulation were designed to observe and compare the dynamics of water penetration into the surface (Figure S33 and S34, Supporting Information). Geometric relaxation of the water was conducted including the surface model, and then it was carried out in an NVT ensemble with a Nose–Hoover thermostat^[^ ^57^ ^]^ for the fully relaxed structure at temperatures greater than 298 K. A time step of 1 fs and total simulation time of 500 ps were used. The total simulation time for each MD simulation was determined using our convergence criteria such as sufficient reaction time and moving velocity of atoms.

## Conflict of Interest

The authors declare no conflict of interest.

## Author Contributions

**Yongju Hong**: conceptualization (lead); formal analysis (equal); visualization (equal); writing—original draft (lead). **Eunsoo Lee**: formal analysis (equal); investigation (lead); visualization (equal); writing—original draft (lead). **Jae Hun Seol**: formal analysis (equal); investigation (lead); visualization (equal); writing—original draft (equal). **Tae Kyung Lee**: formal analysis (equal). **Songa Choi**: formal analysis (supporting). **Seong Chan Cho**: formal analysis (supporting); writing—original draft (supporting). **Taekyung Kim**: formal analysis (supporting). **Hionsuck Baik**: formal analysis (supporting); supervision (supporting). **Sangyeon Jeong**: investigation (supporting). **Sung Jong Yoo**: supervision (equal). **Sang Uck Lee**: supervision (equal). **Kwangyeol Lee**: supervision (lead); writing—original draft (lead). **Yongju Hong**, **Eunsoo Lee**, **Jae Hun Seol**, and **Tae Kyung Lee** contributed equally to this work.

## Supporting information

Supplementary Material

## Data Availability

The data that support the findings of this study are available from the corresponding author upon reasonable request.

## References

[1] Q. Xu , L. Zhang , J. Zhang , J. Wang , Y. Hu , H. Jiang , C. Li , EnergyChem 2022, 4, 100087.

[2] J. F. Callejas , C. G. Read , C. W. Roske , N. S. Lewis , R. E. Schaak , Chem. Mater. 2016, 28, 6017.

[3] Y. Han , Y. Chen , R. Fan , Z. Li , Z. Zou , EcoMat 2021, 3, e12097.

[4] T. Dai , Z. Zhou , H. Xiao , Y. Luo , Y. Xu , X. Wang , Catalysts 2022, 12, 701.

[5] Y. Men , P. Li , J. Zhou , S. Chen , W. Luo , Cell Rep. Phys. Sci. 2020, 1, 100136.

[6] R. Mohili , N. R. Hemanth , H. Jin , K. Lee , N. Chaudhari , J. Mater. Chem. A 2023, 11, 10463.

[7] J. Lee , N. Son , J. Shin , S. Pandey , S. Woo Joo , M. Kang , J. Alloys Compd. 2021, 869, 159265.

[8] Q. Chang , J. Ma , Y. Zhu , Z. Li , D. Xu , X. Duan , W. Peng , Y. Li , G. Zhang , F. Zhang , X. Fan , ACS Sustainable Chem. Eng. 2018, 6, 6388.

[9] J.‐Q. Chi , W.‐K. Gao , J.‐H. Lin , B. Dong , K.‐L. Yan , J.‐F. Qin , B. Liu , Y.‐M. Chai , C.‐G. Liu , ChemSusChem 2018, 11, 743. 29240294 10.1002/cssc.201702010

[10] J.‐C. Kim , C. W. Lee , D.‐W. Kim , J. Mater. Chem. A 2020, 8, 5655.

[11] T. Liu , J. Wang , C. Zhong , S. Lu , W. Yang , J. Liu , W. Hu , C. M. Li , Chem. – Eur. J. 2019, 25, 7826. 30990231 10.1002/chem.201901215

[12] Z. Liu , Z. Li , J. Li , J. Xiong , S. Zhou , J. Liang , W. Cai , C. Wang , Z. Yang , H. Cheng , J. Mater. Chem. A 2019, 7, 5621.

[13] Y. Li , X. Liu , J. Xu , S. Chen , Small 2024, 20, 2402846. 10.1002/smll.202402846 39072957

[14] O. Diaz‐Morales , I. Ledezma‐Yanez , M. T. M. Koper , F. Calle‐Vallejo , ACS Catal. 2015, 5, 5380.

[15] S.‐Y. Bae , J. Mahmood , I.‐Y. Jeon , J.‐B. Baek , Nanoscale Horiz. 2020, 5, 43.

[16] K. Bhunia , M. Chandra , S. Kumar Sharma , D. Pradhan , S.‐J. Kim , Coord. Chem. Rev. 2023, 478, 214956.

[17] Y. Hong , S. C. Cho , S. Kim , H. Jin , J. H. Seol , T. K. Lee , J.‐K. Ryu , G. M. Tomboc , T. Kim , H. Baik , C. Choi , J. Jo , S. Jeong , E. Lee , Y. Jung , D. Ahn , Y.‐T. Kim , S. J. Yoo , S. U. Lee , K. Lee , Adv. Energy Mater. 2024, 14, 2304269.

[18] Y. Liu , P. Vijayakumar , Q. Liu , T. Sakthivel , F. Chen , Z. Dai , Nano‐Micro Lett. 2022, 14, 43. 10.1007/s40820-021-00785-2 PMC8724338 34981288

[19] D. Chen , J. Zhu , Z. Pu , S. Mu , Chem. Eur. J. 2021, 27, 12257. 34129268 10.1002/chem.202101645

[20] Y. Li , J. Chen , Z. A. Wang , S. Chen , ACS Appl. Nano Mater. 2024, 7, 7555.

[21] E. Lee , S. Jeong , Y. Jeong , B. Kim , K. Lee , Small Methods 2025, 9, 2301782. 10.1002/smtd.202301782 38775629

[22] J. Zhu , S. Li , M. Xiao , X. Zhao , G. Li , Z. Bai , M. Li , Y. Hu , R. Feng , W. Liu , R. Gao , D. Su , A. Yu , Z. Chen , Nano Energy 2020, 77, 105212.

[23] M. A. R. Anjum , J. S. Lee , ACS Catal. 2017, 7, 3030.

[24] M. W. Khan , S. Loomba , R. Ali , M. Mohiuddin , A. Alluqmani , F. Haque , Y. Liu , R. U. R. Sagar , A. Zavabeti , T. Alkathiri , B. Shabbir , X. Jian , J. Z. Ou , A. Mahmood , N. Mahmood , Front. Chem. 2020, 8, 733. 33005605 10.3389/fchem.2020.00733 PMC7484372

[25] Y. Park , M. Jun , T. Kwon , J. Y. Kim , K. Lee , Chem Catal. 2023, 3, 100499.

[26] C. An , Y. Wang , P. Jiao , S. Wu , L. Gao , C. Zhu , J. Li , N. Hu , Catalysts 2022, 12, 1055.

[27] Y.‐C. Chu , C.‐J. Chang , Y. Zhu , S.‐C. Lin , C.‐W. Tung , T.‐L. Chen , H. M. Chen , ACS Sustainable Chem. Eng. 2019, 7, 14247.

[28] S. Jo , C. H. Lee , H. Jin , E. Lee , T. Kim , H. Baik , S. U. Lee , S. J. Yoo , K. Lee , J. Park , ACS Nano 2024, 18, 15705. 38848500 10.1021/acsnano.4c01997

[29] Y. Hong , T. Kim , J. Jo , B. Kim , H. Jin , H. Baik , K. Lee , ACS Nano 2020, 14, 11205. 32628443 10.1021/acsnano.0c02891

[30] Y. Hong , S. Jeong , J. H. Seol , T. Kim , S. C. Cho , T. K. Lee , C. Yang , H. Baik , H. S. Park , E. Lee , S. J. Yoo , S. U. Lee , K. Lee , Adv. Energy Mater. 2024, 14, 2401426.

[31] R. W. Lord , J. Fanghanel , C. F. Holder , I. Dabo , R. E. Schaak , Chem. Mater. 2020, 32, 10227.

[32] W. H. Lee , Y.‐J. Ko , J. H. Kim , C. H. Choi , K. H. Chae , H. Kim , Y. J. Hwang , B. K. Min , P. Strasser , H.‐S. Oh , Nat. Commun. 2021, 12, 4271. 34257287 10.1038/s41467-021-24578-8 PMC8277764

[33] Y. Li , L. A. Zhang , Y. Qin , F. Chu , Y. Kong , Y. Tao , Y. Li , Y. Bu , D. Ding , M. Liu , ACS Catal. 2018, 8, 5714.

[34] Z. Wang , N. Heng , X. Wang , J. He , Y. Zhao , J. Catal. 2019, 374, 51.

[35] I.‐S. Kim , H.‐S. Cho , M. Kim , H.‐J. Oh , S.‐Y. Lee , Y.‐K. Lee , C. Lee , J. H. Lee , W. C. Cho , S.‐K. Kim , J. H. Joo , C.‐H. Kim , J. Mater. Chem. A 2021, 9, 16713.

[36] Y. Wang , Z. Liu , H. Liu , N.‐T. Suen , X. Yu , L. Feng , ChemSusChem 2018, 11, 2724. 29888872 10.1002/cssc.201801103

[37] J. Kibsgaard , T. F. Jaramillo , Angew. Chem., Int. Ed. 2014, 53, 14433. 10.1002/anie.201408222 25359678

[38] J. Kibsgaard , C. Tsai , K. Chan , J. D. Benck , J. K. Nørskov , F. Abild‐Pedersen , T. F. Jaramillo , Energy Environ. Sci. 2015, 8, 3022.

[39] R. Miao , B. Dutta , S. Sahoo , J. He , W. Zhong , S. A. Cetegen , T. Jiang , S. P. Alpay , S. L. Suib , J. Am. Chem. Soc. 2017, 139, 13604. 28871790 10.1021/jacs.7b07044

[40] Y. Zhu , M. Klingenhof , C. Gao , T. Koketsu , G. Weiser , Y. Pi , S. Liu , L. Sui , J. Hou , J. Li , H. Jiang , L. Xu , W.‐H. Huang , C.‐W. Pao , M. Yang , Z. Hu , P. Strasser , J. Ma , Nat. Commun. 2024, 15, 1447. 38365760 10.1038/s41467-024-45654-9 PMC10873302

[41] X. Chen , X.‐T. Wang , J.‐B. Le , S.‐M. Li , X. Wang , Y.‐J. Zhang , P. Radjenovic , Y. Zhao , Y.‐H. Wang , X.‐M. Lin , J.‐C. Dong , J.‐F. Li , Nat. Commun. 2023, 14, 5289. 37648700 10.1038/s41467-023-41030-1 PMC10468501

[42] J. K. Nørskov , J. Rossmeisl , A. Logadottir , L. Lindqvist , J. R. Kitchin , T. Bligaard , H. Jónsson , J. Phys. Chem. B 2004, 108, 17886. 39682080 10.1021/jp047349j

[43] I. C. Man , H.‐Y. Su , F. Calle‐Vallejo , H. A. Hansen , J. I. Martínez , N. G. Inoglu , J. Kitchin , T. F. Jaramillo , J. K. Nørskov , J. Rossmeisl , ChemCatChem 2011, 3, 1159.

[44] C. H. Lee , B. Jun , S. U. Lee , ACS Sustainable Chem. Eng. 2018, 6, 4973.

[45] B. Hammer , J. K. Norskov , Nature 1995, 376, 238.

[46] J. Mahmood , F. Li , S.‐M. Jung , M. S. Okyay , I. Ahmad , S.‐J. Kim , N. Park , H. Y. Jeong , J.‐B. Baek , Nat. Nanotechnol. 2017, 12, 441. 28192390 10.1038/nnano.2016.304

[47] Y. Zuo , S. Bellani , M. Ferri , G. Saleh , D. V. Shinde , M. I. Zappia , R. Brescia , M. Prato , L. De Trizio , I. Infante , F. Bonaccorso , L. Manna , Nat. Commun. 2023, 14, 4680. 37542064 10.1038/s41467-023-40319-5 PMC10403570

[48] G. Kresse , J. Furthmüller , Comput. Mater. Sci. 1996, 6, 15.

[49] G. Kresse , J. Furthmüller , Phys. Rev. B: Condens. Matter 1996, 54, 11169. 9984901 10.1103/physrevb.54.11169

[50] P. E. Blöchl , Phys. Rev. B 1994, 50, 17953. 10.1103/physrevb.50.17953 9976227

[51] G. Kresse , D. Joubert , Phys. Rev. B 1999, 59, 1758.

[52] J. P. Perdew , K. Burke , M. Ernzerhof , Phys. Rev. Lett. 1997, 78, 1396. 10.1103/PhysRevLett.77.3865 10062328

[53] H. J. Monkhorst , J. D. Pack , Phys. Rev. B 1976, 13, 5188.

[54] S. Grimme , J. Antony , S. Ehrlich , H. Krieg , J. Chem. Phys. 2010, 132, 154104. 20423165 10.1063/1.3382344

[55] S. R. Kelly , C. Kirk , K. Chan , J. K. Nørskov , J. Phys. Chem. C 2020, 124, 14581.

[56] A. K. Rappe , C. J. Casewit , K. S. Colwell , W. A. Goddard III , W. M. Skiff , J. Am. Chem. Soc. 1992, 114, 10024.

[57] N. Shuichi , Prog. Theor. Phys. Suppl. 1991, 103, 1.

