# Supplementary material for: Se‐Assisted Modulation of Electronic Structure of Ruthenium Phosphide Nanotubes for Efficient Alkaline Hydrogen Evolution Reaction
Source: Small Sci. 2025 Mar 13;5(6):2400610. doi: 10.1002/smsc.202400610 (PMC12168615; doi:10.1002/smsc.202400610)
Supplement: Supplementary file 1 — Supplementary Material [file SMSC-5-2400610-s001.pdf]

Supporting Information

Se-assisted Modulation of Electronic Structure of Ruthenium Phosphide Nanotubes for Efficient Alkaline Hydrogen Evolution Reaction

Yongju Hong[a,b,#], Eunsoo Lee[a,#], Jae Hun Seol[c,#], Tae Kyung Lee[b,#], Songa. Choi[a], Seong Chan Cho[c], Taekyung Kim[d], Hionsuck Baik[d], Sangyeon Jeong[a], Sung Jong Yoo[b,g,h*], Sang Uck Lee[c,*], and Kwangyeol Lee[a,*]

a Department of Chemistry and Research Institute for Natural Sciences, Korea University, Seoul 02841, Republic of Korea
E-mail: kylee1@korea.ac.kr
b Hydrogen·Fuel Cell Research Center, Korea Institute of Science and Technology (KIST), Seoul 02792, Republic of Korea
E-mail: ysj@kist.re.kr
c School of Chemical Engineering, Sungkyunkwan University, Suwon 16419, Republic of Korea
E-mail: suleechem@skku.edu
d Korea Basic Science Institute (KBSI), Seoul 02841, Republic of Korea
g Division of Energy & Environment Technology, KIST School, University of Science and Technology (UST), Daejeon 34113, Republic of Korea
h KHU-KIST Department of Converging Science and Technology, Kyung Hee University, Seoul 02447, Republic of Korea

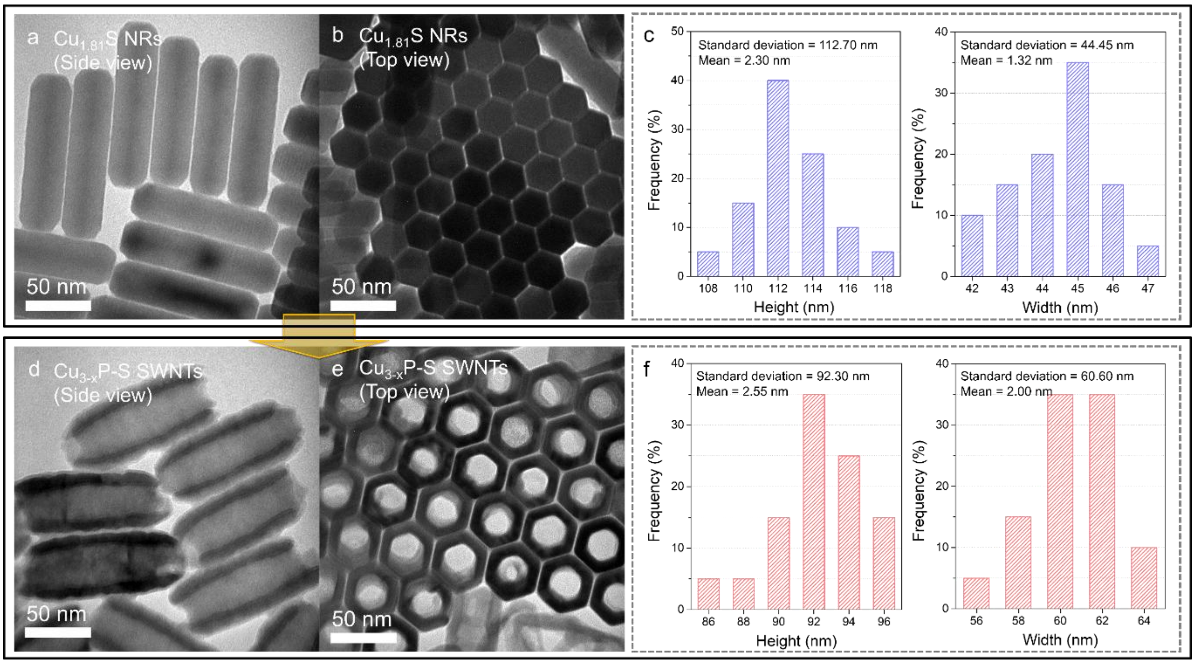

Figure S1. Representative top and side–view TEM images with corresponding histograms for height and width distributions. (a–c) roxbyite $Cu_{1.81}S$ nanorods (CS NRs) and (d–f) trigonal $Cu_{3-x}P$–S single–walled nanotubes (CPS SWNTs).

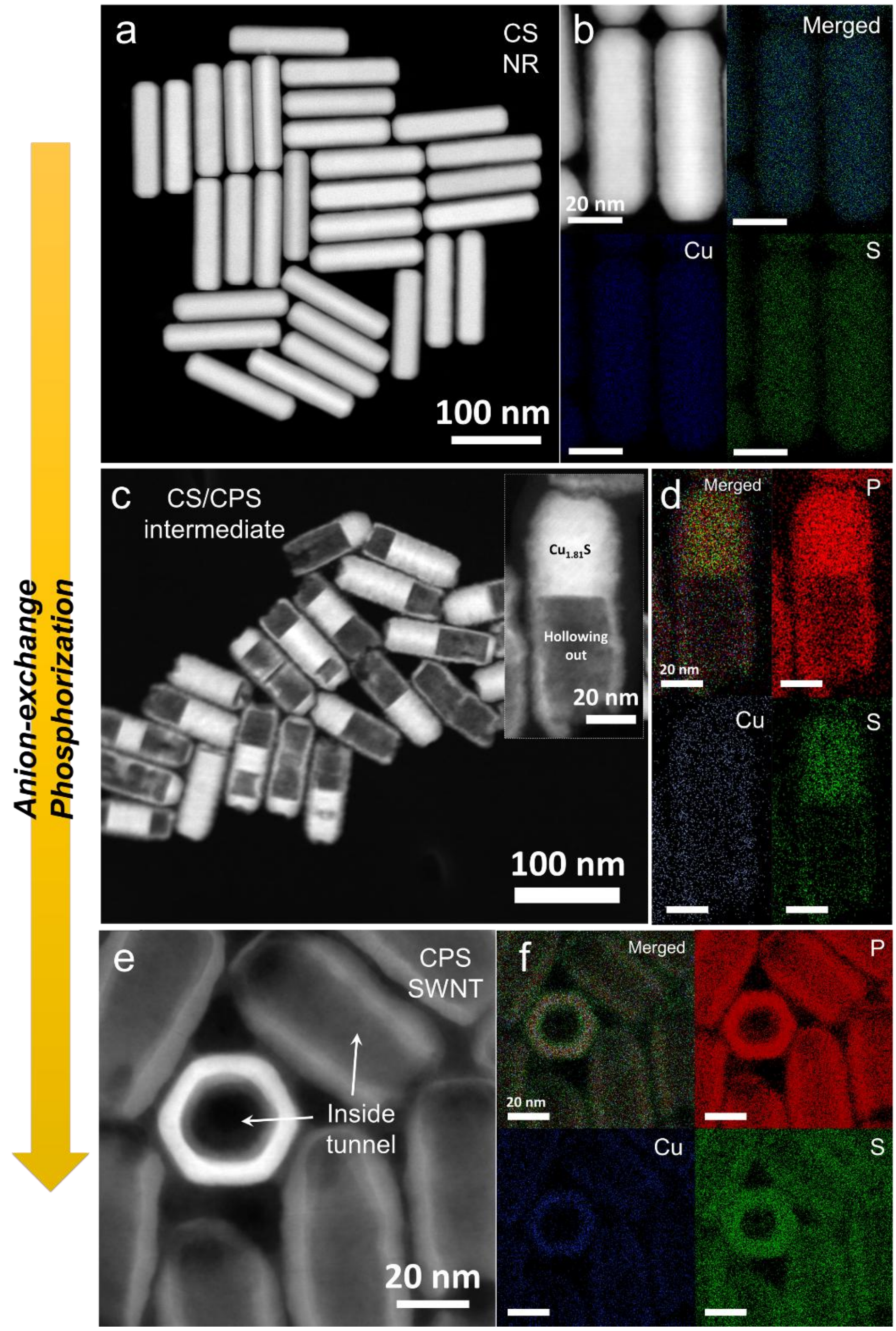

Figure S2. Topotactic transformation from CS NR to CPS SWNT followed by anion exchange reaction. STEM and corresponding EDS elemental mapping images of the CS NR (a-b), half–proceeded intermediates (c-d), and CPS SWNT (e-f), respectively. STEM image of the intermediates shows the hollowing process as a result of the anion exchange–induced Kirkendall effect.

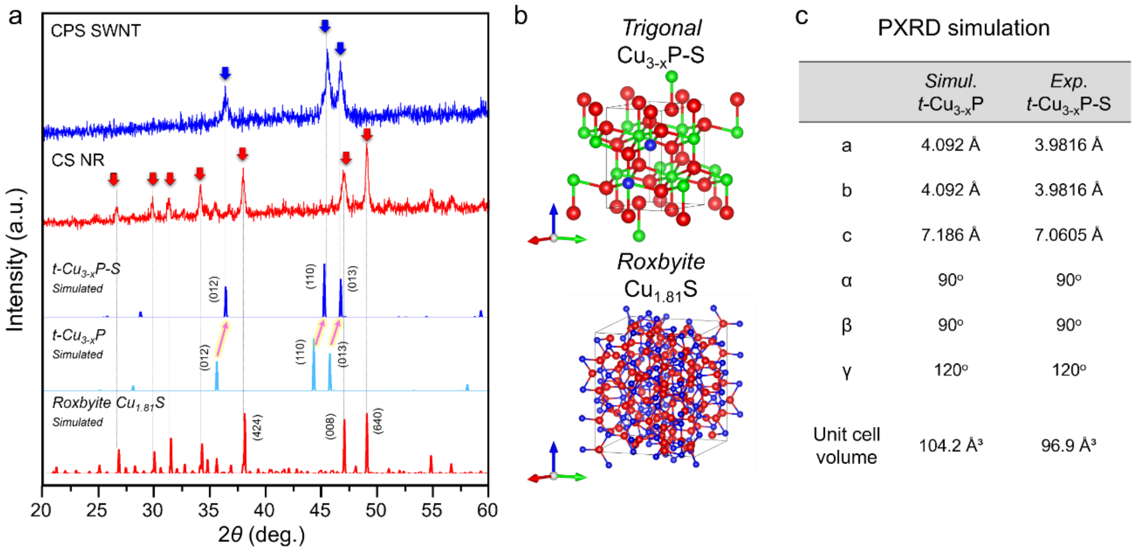

Figure S3. (a) PXRD patterns of CS NRs and CPS SWNTs in comparison with roxbyite $Cu_{1.81}S$ (PDF# 00–064–0278), trigonal $Cu_{3-x}P$ (*t*–$Cu_{3-x}P$) phase (PDF# 01–072–0807), and simulated *t*–$Cu_{3-x}P$–S, of which the unit cell parameters are calculated, considering the substitution of S with P. (b) The crystal structures of CS and *t*–$Cu_{3-x}P$–S (c) The unit cell parameters of PXRD pattern in (a), which indicates that CPS SWNT inherits a *t*–$Cu_{3-x}P$ crystal structure with smaller crystal lattice parameters.

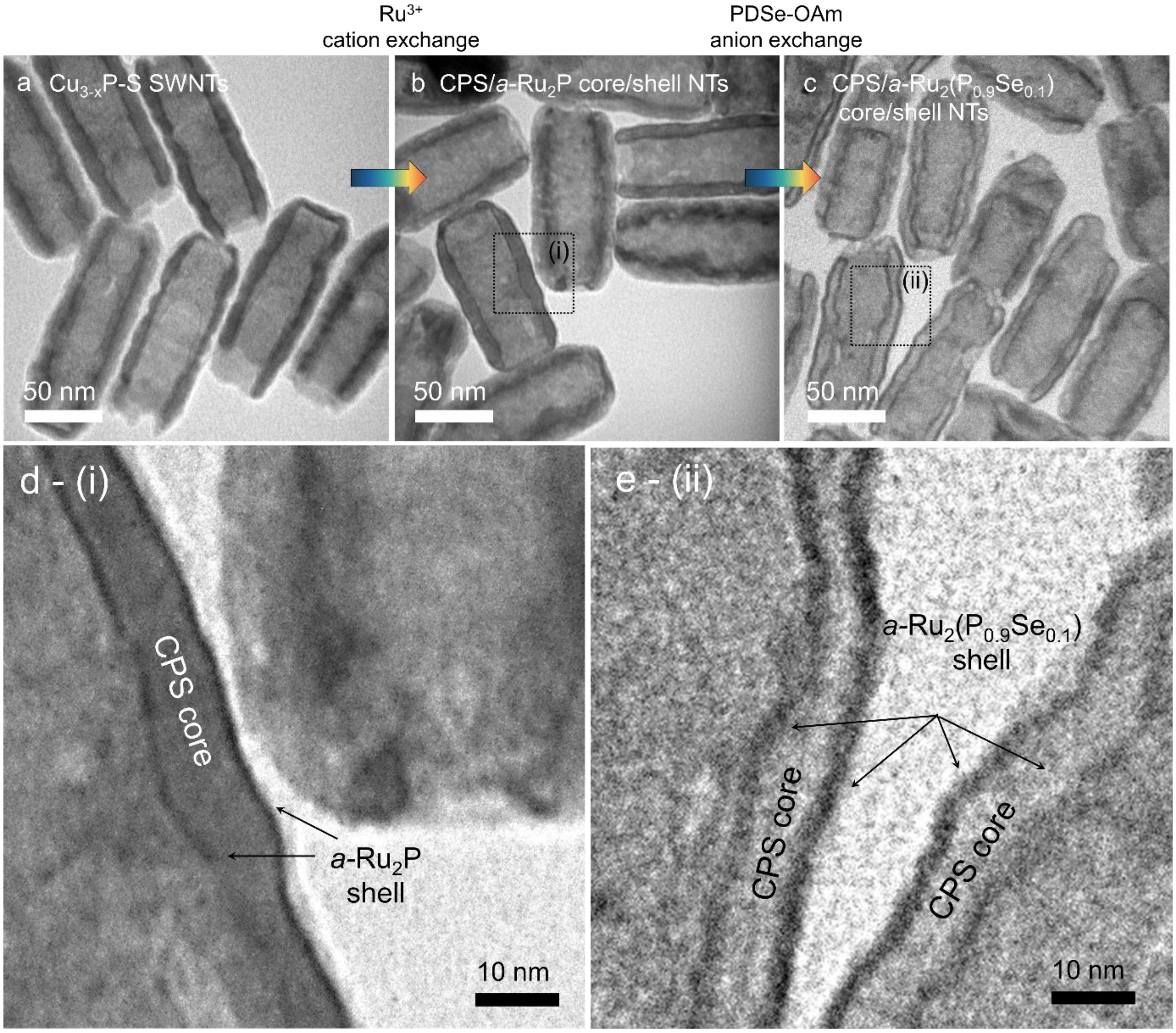

Figure S4. Topotactic transformation from CPS SWNT to CPS/*a*–$Ru_2(P_{0.9}Se_{0.1})$ followed by cation and anion exchange. TEM images of the (a) CPS SWNT, (b) CPS/*a*–Ru_2P core/shell NTs, and (c) CPS/*a*–$Ru_2(P_{0.9}Se_{0.1})$ core/shell NTs. (d) Magnified TEM image of (i) area in fig(b). CPS core and *a*–Ru_2P shell appear in contrast, and the formation of *a*–Ru_2P shell can be clearly identified. (e) Magnified TEM image of (ii) area in fig(c).

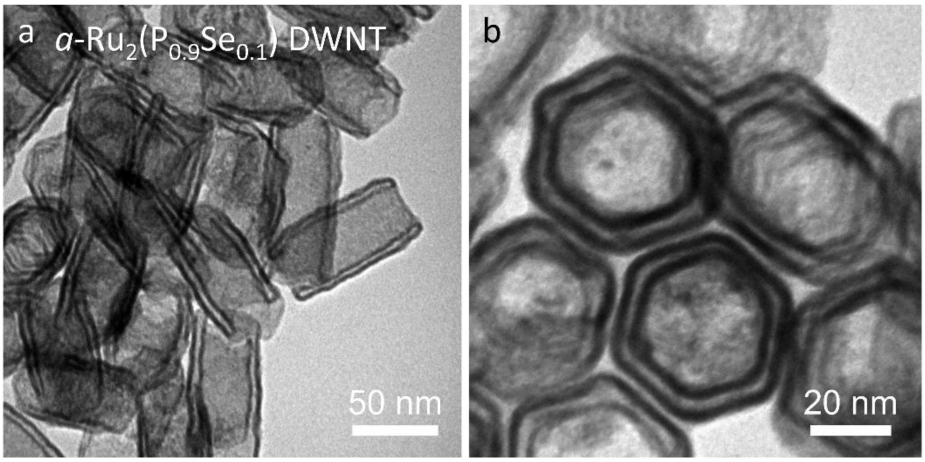

Figure S5. Representative (a) top and (b) side–view TEM images of *a*–$Ru_2(P_{0.9}Se_{0.1})$ DWNTs.

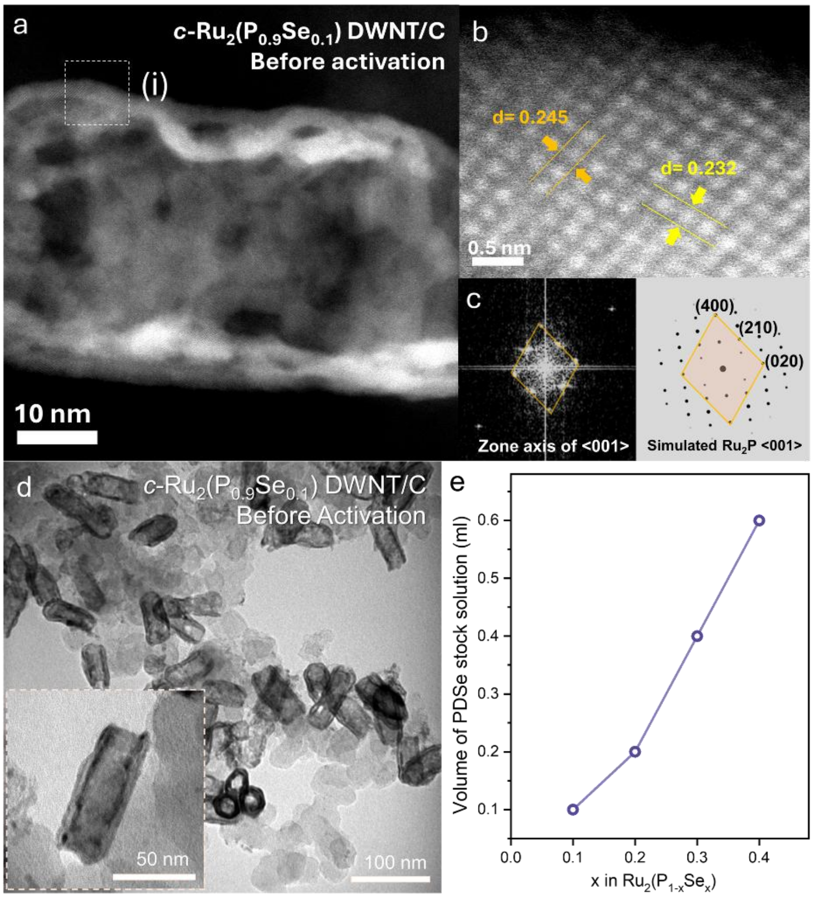

Figure S6. TEM images and compositional analysis of *c*–$Ru_2(P_{0.9}Se_{0.1})$ DWNT/C after calcination. Representative (a) STEM image and (b) atomic-scale resolution STEM image with corresponding (c) FFT analysis, and (d) TEM images of *c*-$Ru_2(P_{0.9}Se_{0.1})$ DWNT/C. (e) The graph showing the correlation between the concentration of the injected PDSe-OAm stock solution and the Se ratio within the generated *c*-$Ru_2(P_{1-x}Se_x)$ DWNTs.

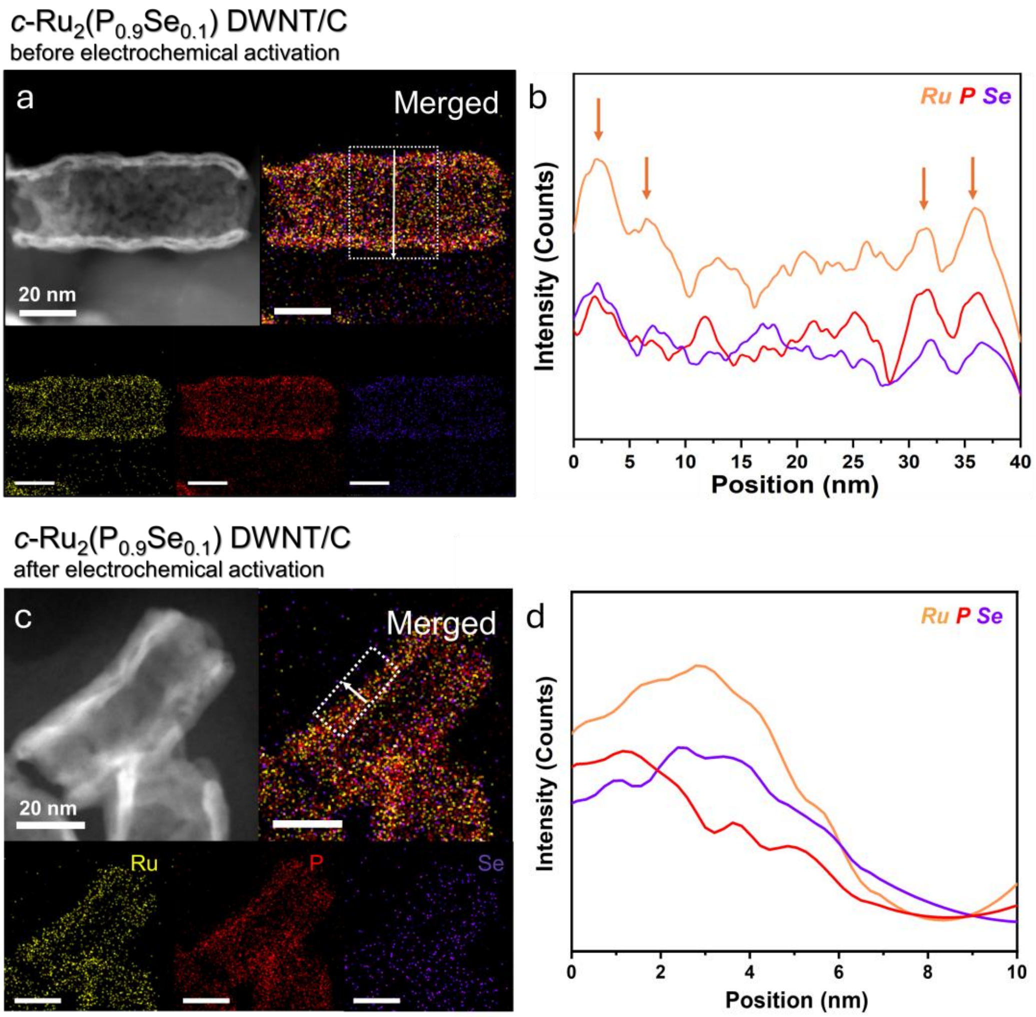

Figure S7. HAADF–STEM image with corresponding elemental mapping images of *c*–$Ru_2(P_{0.9}Se_{0.1})$ DWNT/C (a) before and (c) after electrochemical treatment. (b, d) The line-profiling analysis along the direction shown in (a) and in (c), respectively.

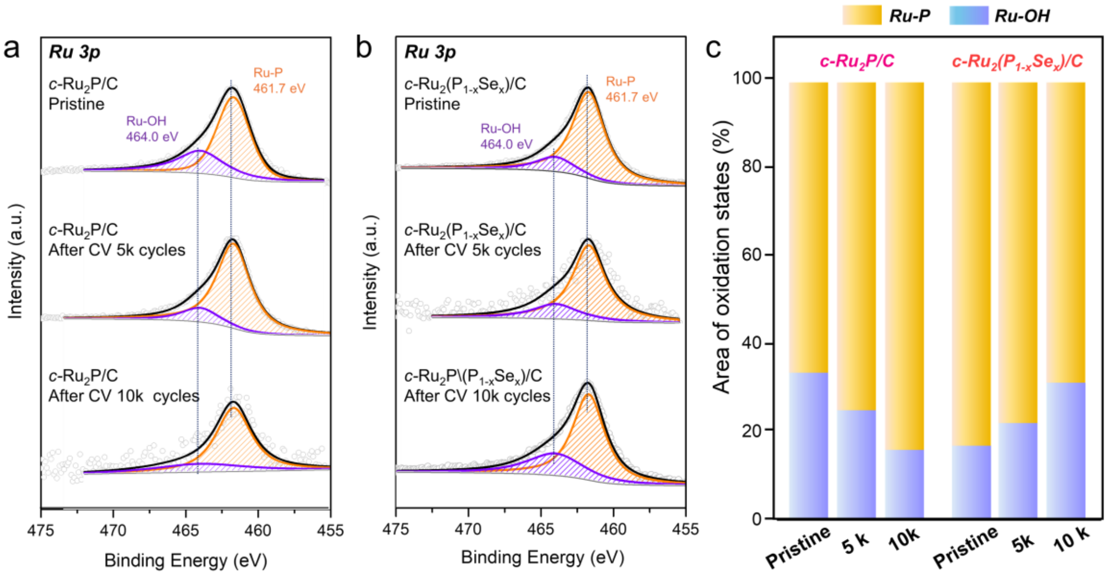

Figure S8. XPS analysis of the Ru 3p region of (a) *c*–Ru_2P and (b) *c*–$Ru_2(P_{0.9}Se_{0.1})$ DWNT/C catalysts during electrochemical treatment cycles of 0k, 5k, and 10k. (c) Corresponding area percentage of deconvoluted Ru 3p XPS of *c*–Ru_2P and *c*–$Ru_2(P_{0.9}Se_{0.1})$ DWNT/C.

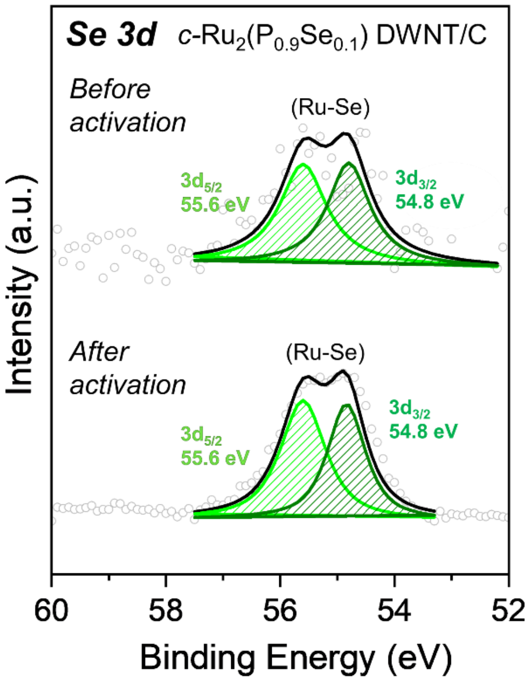

Figure S9. XPS analysis of the Se 3d region of pristine and activated *c*–$Ru_2(P_{0.9}Se_{0.1})$ DWNT/C.

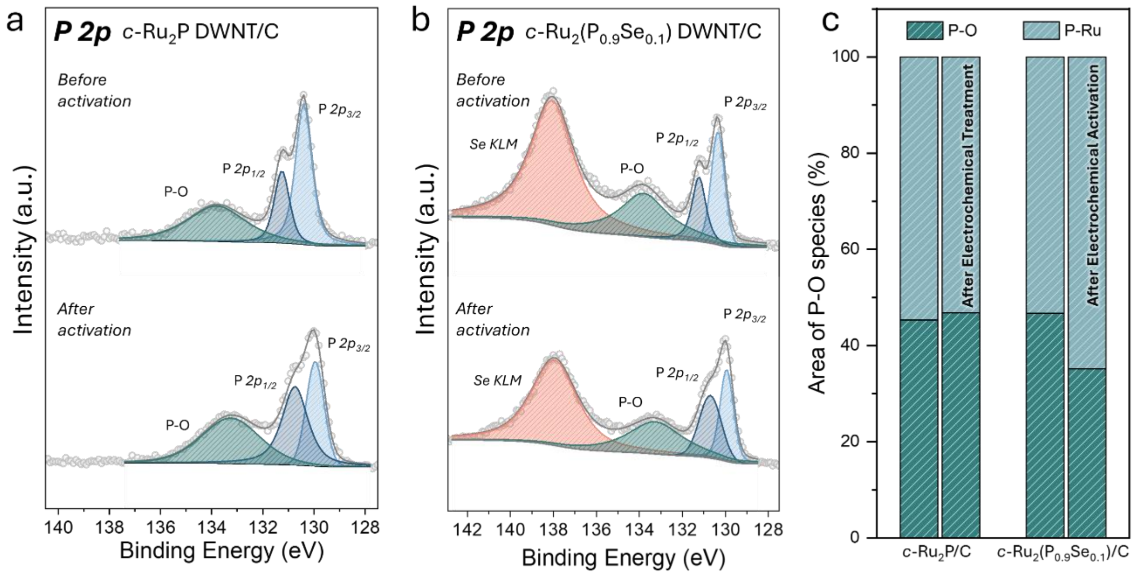

Figure S10. XPS analysis of the P 2p region of (a) *c*–Ru_2P and (b) *c*–$Ru_2(P_{0.9}Se_{0.1})$ DWNT/C before and after electrochemical treatment/activation. (c) Corresponding area percentage of deconvoluted P 2p XPS of *c*–Ru_2P and *c*–$Ru_2(P_{0.9}Se_{0.1})$ DWNT/C.

Note S1. X-ray photoelectron spectroscopy (XPS) was carried out to probe the electronic states of P in *c*–Ru_2P and *c*–$Ru_2(P_{0.9}Se_{0.1})$ DWNT/C. The XPS spectra of P 2p regions revealed characteristic metal-P bonds (130.4 eV for P $2p_{3/2}$, 131.2 eV for P $2p_{1/2}$) with oxidized species (P-O) at 133.3 eV. [1] After electrochemical activation, the percentage of oxidized species (P-O) in *c*–$Ru_2(P_{0.9}Se_{0.1})$ DWNT/C was reduced, which can be attributed to the formation of Ru-OH species. In contrast, negligible changes were observed in *c*–Ru_2P DWNT/C. [2]

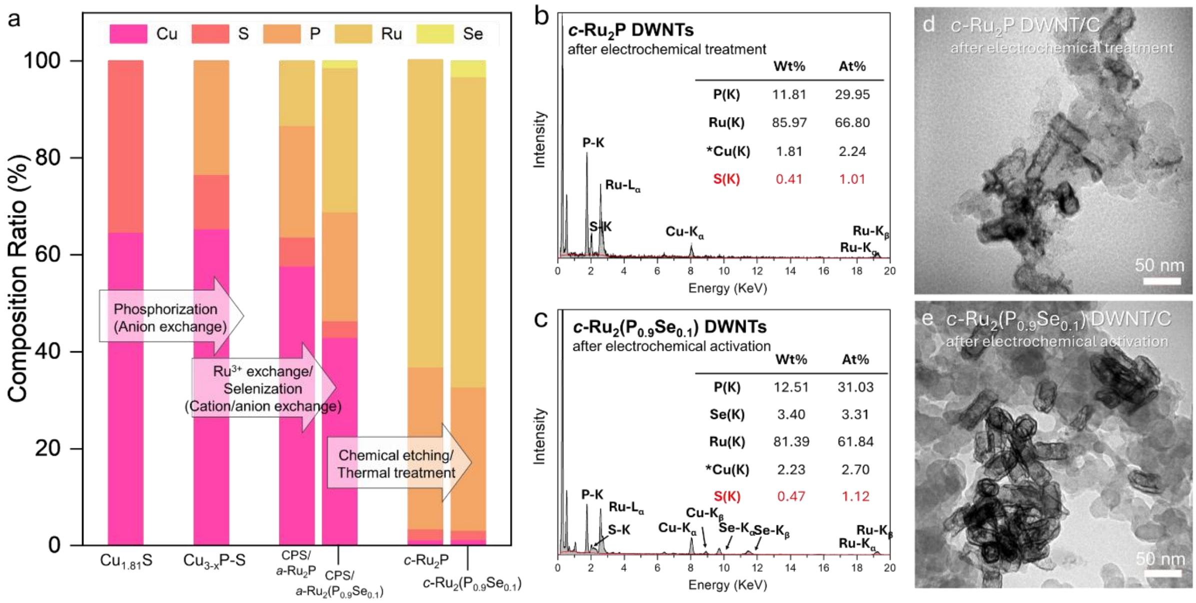

Figure S11. (a) Compositional analysis of EDS data results for CS NR, CPS SWNT, CPS/*a*–Ru_2P core/shell NT, CPS/*a*–$Ru_2(P_{0.9}Se_{0.1})$ core/shell NT, *c*–Ru_2P DWNT/C, and activated *c*–$Ru_2(P_{0.9}Se_{0.1})$ DWNT/C. (b, c) EDS spectra with corresponding elemental analysis data. (The measured sulfur (S) content, which is below 1%, is negligible, and the detected copper (Cu) is presumed to be due to the TEM holder.) (d, e) TEM images for treated *c*–Ru_2P DWNT/C and activated *c*–$Ru_2(P_{0.9}Se_{0.1})$ DWNT/C, respectively.

ICP-OES							
Catalysts		Weight Percent (%)			Atomic Percent (%)		
		Ru	P	Se	Ru	P	Se
Before electrochemical treatment/activation	*c*-Ru_2P DWNT/C	88.11	11.89	-	69.42	30.58	-
	c-$Ru_2(P_{0.9}Se_{0.1})$ DWNT/C	83.84	12.74	3.52	64.49	32.04	3.47
After electrochemical treatment/activation	*c*-Ru_2P DWNT/C	85.88	14.12	-	65.09	34.91	-
	c-$Ru_2(P_{0.9}Se_{0.1})$ DWNT/C	84.66	11.99	3.35	66.10	30.55	3.35

Figure S12. ICP-OES data of *c*–Ru_2P and *c*–$Ru_2(P_{0.9}Se_{0.1})$ DWNT/C before and after electrochemical treatment/activation.

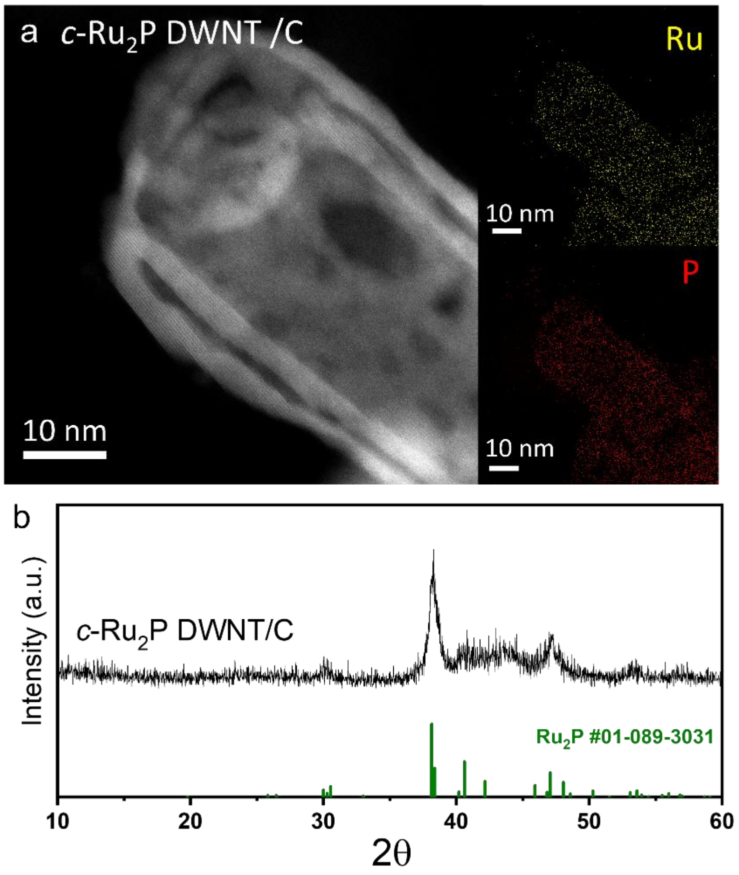

Figure S13. HRTEM and compositional analysis of *c*–Ru_2P DWNT/C. (a) HR-STEM image from the side–edge view with corresponding elemental mapping image. (b) PXRD pattern of *c*-Ru_2P DWNT/C. Colored sticks in PXRD patterns indicate references: Ru_2P (PDF#01-089-3031).

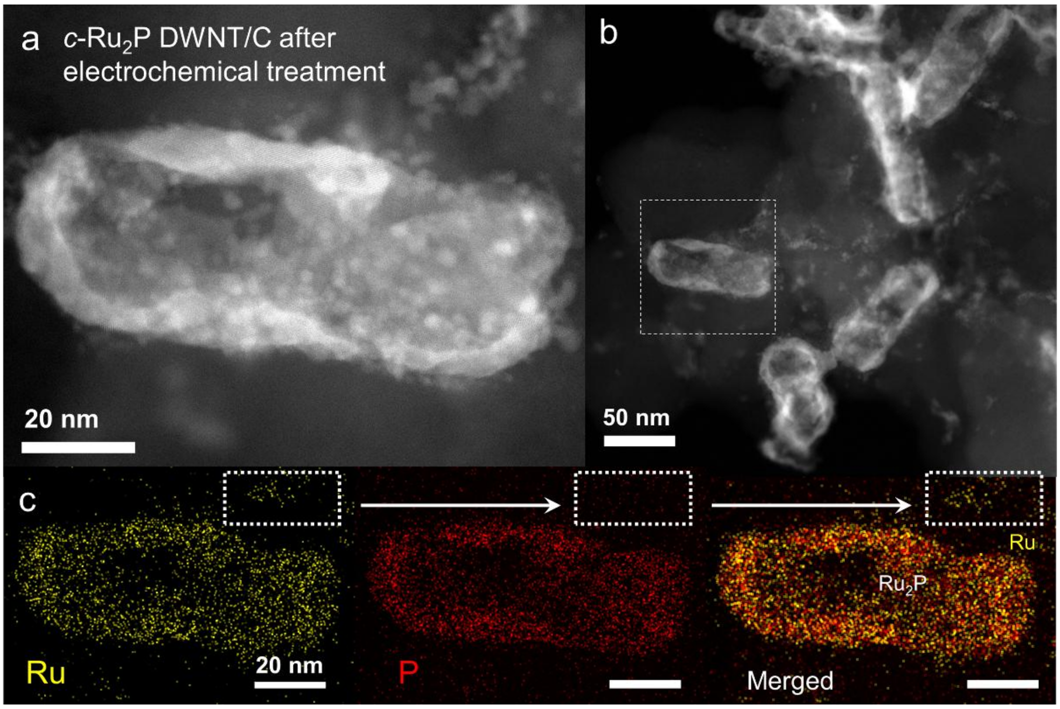

Figure S14. HAADF–STEM and compositional analysis of *c*–Ru_2P DWNT/C after electrochemical treatment. (a) HAADF–STEM image from the side–edge view and (b) low-resolution. (c) EDS elemental mapping images of *c*–Ru_2P DWNT/C after electrochemical treatment.

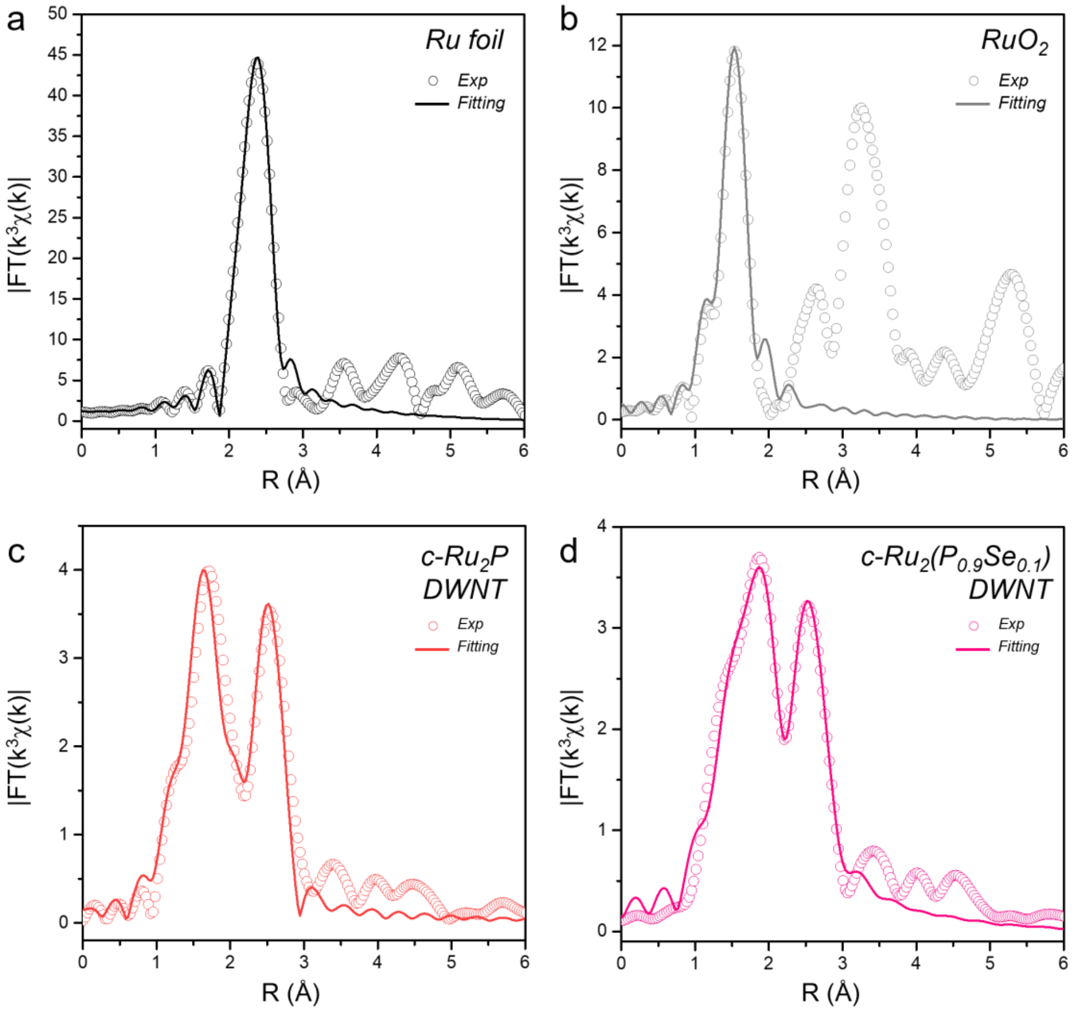

Figure S15. Fitting results of FT–EXAFS for (a) Ru foil, (b) RuO_2, (c) treated *c*–Ru_2P DWNTs, and (d) activated *c*–$Ru_2(P_{0.9}Se_{0.1})$ DWNTs.

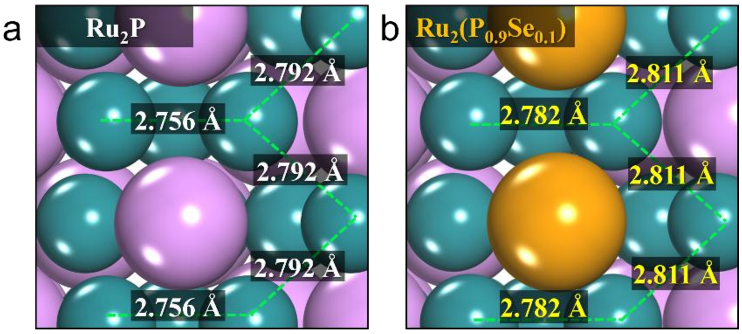

Figure S16. Simulated crystal models of (a) *c*–$Ru_2(P_{0.9}Se_{0.1})$ and (b) pristine Ru_2P with expected lattice parameters.

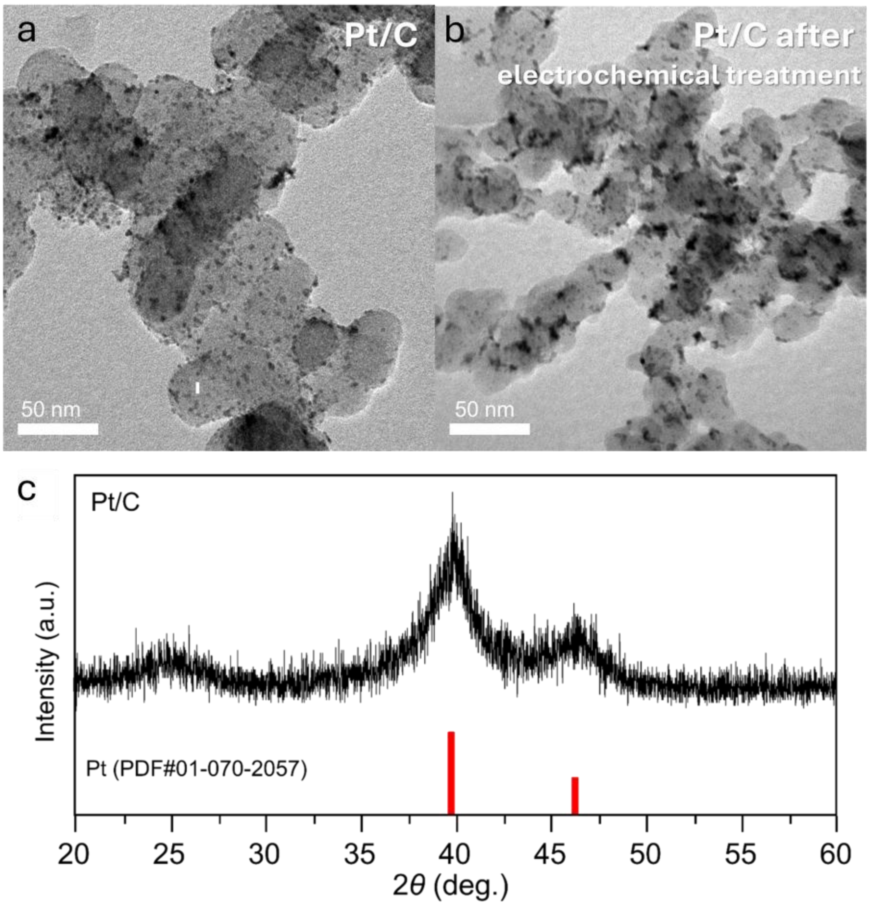

Figure S17. (a) TEM images of commercial Pt/C and (b) after electrochemical treatment. (c) PXRD patterns of Pt/C. Colored sticks in PXRD patterns indicate references: Pt (PDF#01-070-2057).

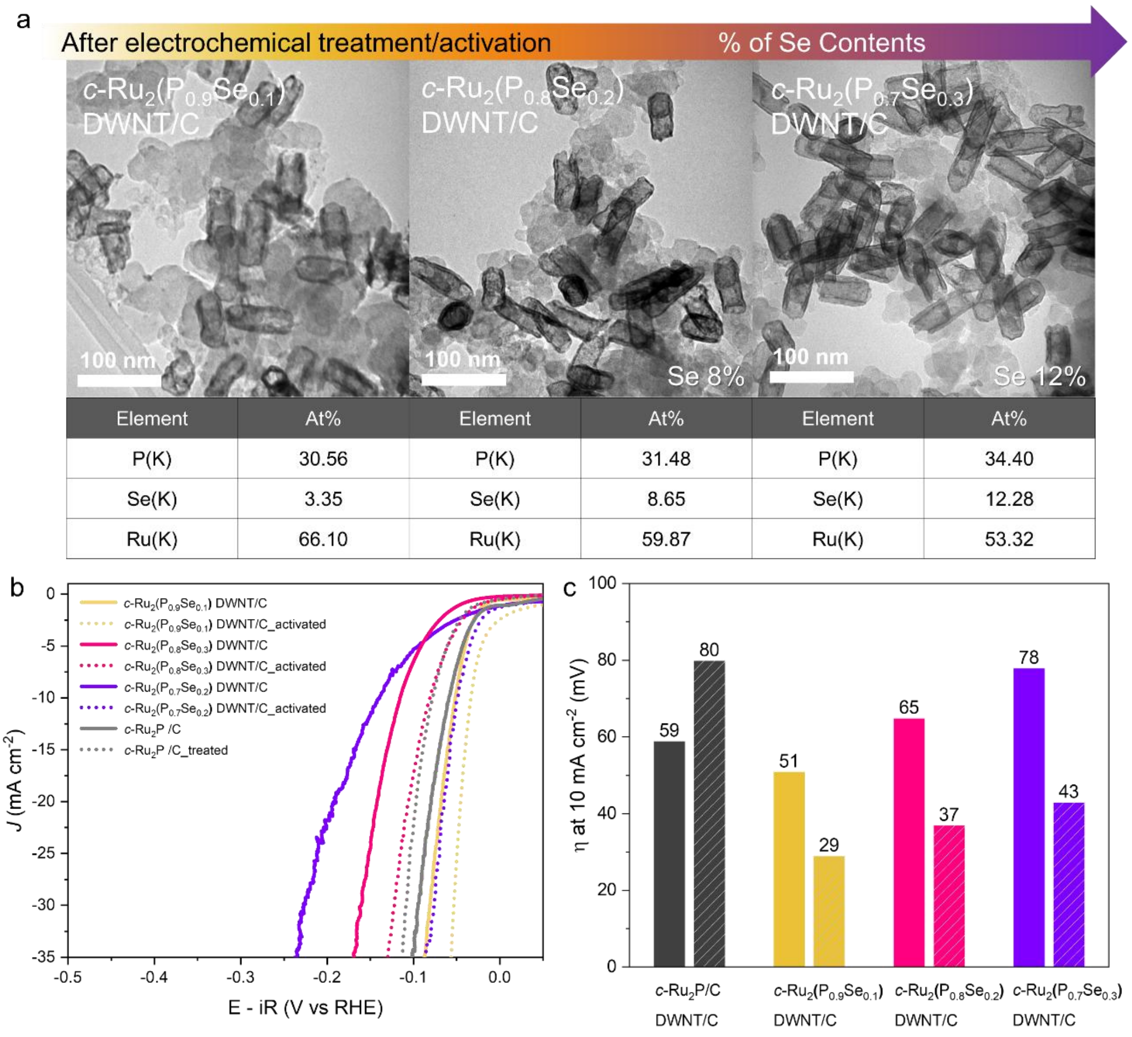

Element	At%	Element	At%	Element	At%
P(K)	30.56	P(K)	31.48	P(K)	34.40
Se(K)	3.35	Se(K)	8.65	Se(K)	12.28
Ru(K)	66.10	Ru(K)	59.87	Ru(K)	53.32

Figure S18. Se-doping level-dependent alkaline HER activities. (a) TEM and EDS analysis of *c*–$Ru_2(P_{0.9}Se_{0.1})$, *c*–$Ru_2(P_{0.8}Se_{0.2})$, and *c*–$Ru_2(P_{0.7}Se_{0.3})$ DWNT/C catalysts after electrochemical treatment/activation. (b) *iR*–corrected HER polarization curves of the catalysts before and after the treatment/activation. (c) Overpotentials of the catalysts derived from HER polarization curves at 10 mA cm^{-2}.

Note S2: The *c*–$Ru_2(P_{0.9}Se_{0.1})$, *c*–$Ru_2(P_{0.8}Se_{0.2})$, and *c*–$Ru_2(P_{0.7}Se_{0.3})$ DWNT/C catalysts were prepared, all with the same structure and size was confirmed in TEM images. Following the electrochemical treatment, all three catalyst types exhibited activation behavior, resulting in decreased overpotential. It is noteworthy that the optimal composition, *c*–$Ru_2(P_{0.9}Se_{0.1})$ DWNT/C, demonstrated superior performance both before and after treatment compared to the other compositions.

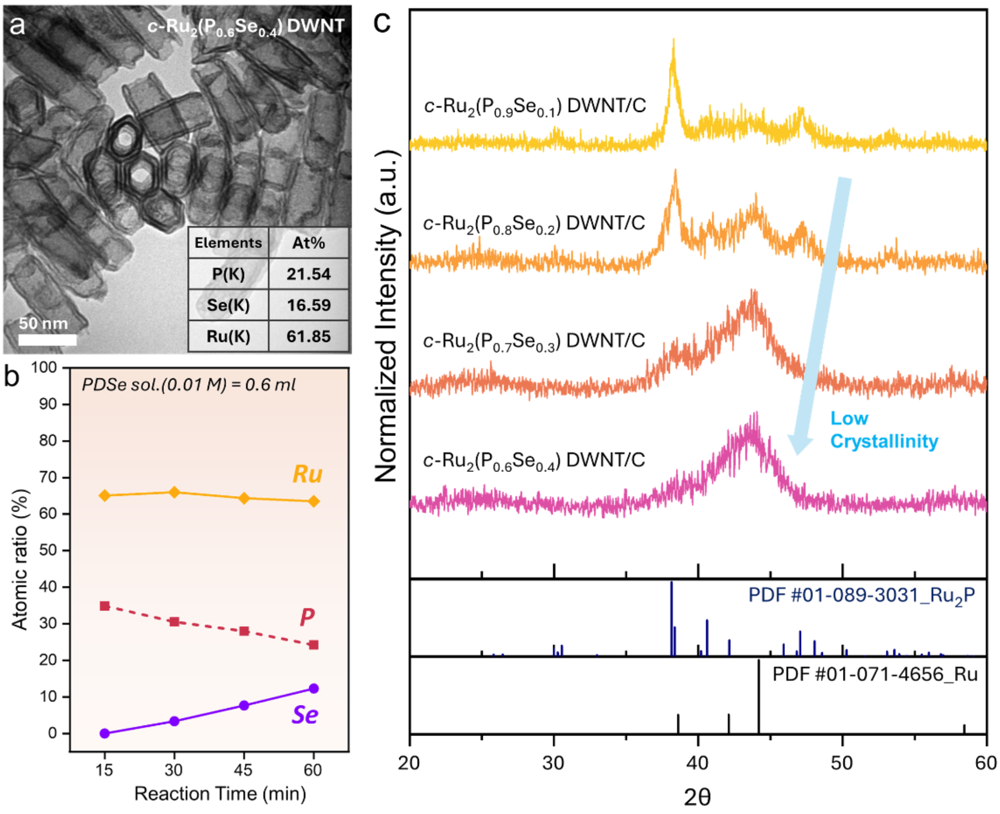

Figure S19. (a) TEM image of *c*–$Ru_2(P_{0.6}Se_{0.4})$ DWNT/C and (b) graph displaying the change in the atomic ratio of Ru, P, and Se over anion exchange reaction time of *c*–$Ru_2(P_{0.6}Se_{0.4})$ DWNT/C synthesis. (c) PXRD patterns of *c*–$Ru_2(P_{1-x}Se_x)$ DWNT/C. The crystallinity of *c*–$Ru_2(P_{1-x}Se_x)$ DWNT/C decreases as the Se ratio increases, leading to reduce HER performances.

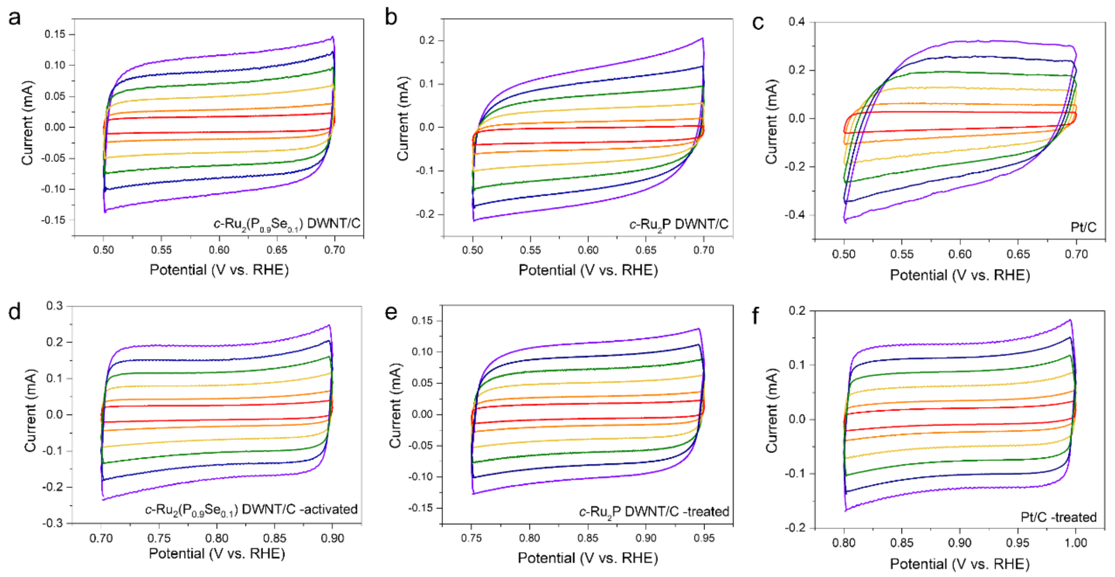

Figure S20. Estimation of the relative electrochemically active surface areas using CV measurements by extracting the double–layer capacitance (C_{dl}). CV curves of *c*–$Ru_2(P_{0.9}Se_{0.1})$ DWNT/C, *c*–Ru_2P DWNT/C, and Pt/C before (a–c) and after electrochemical treatment (d–f) with scan rates from 10 to 160 mV s^{-1}. ,

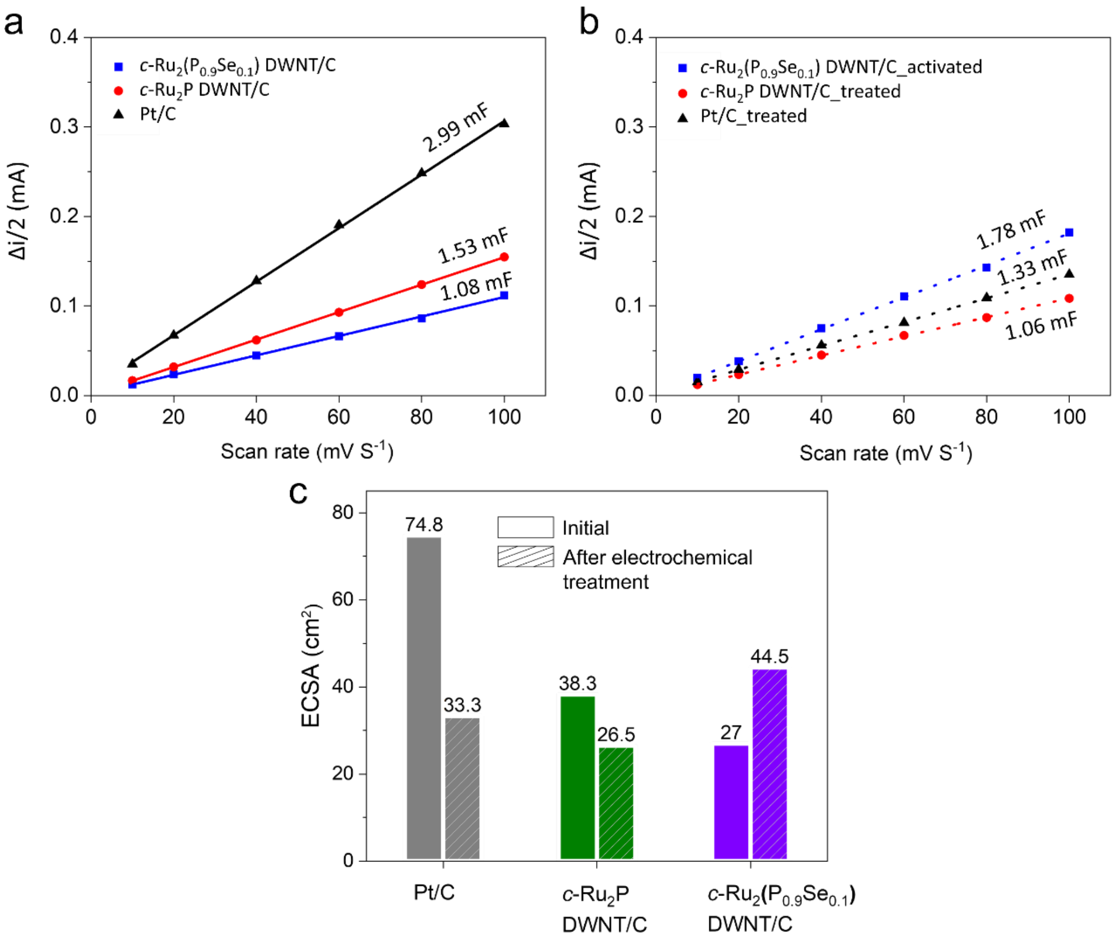

Figure S21. (a,b) Linear plots of capacitive current against scan rate before and after electrochemical treatment. (c) Bar graphs of ECSA for each catalyst.

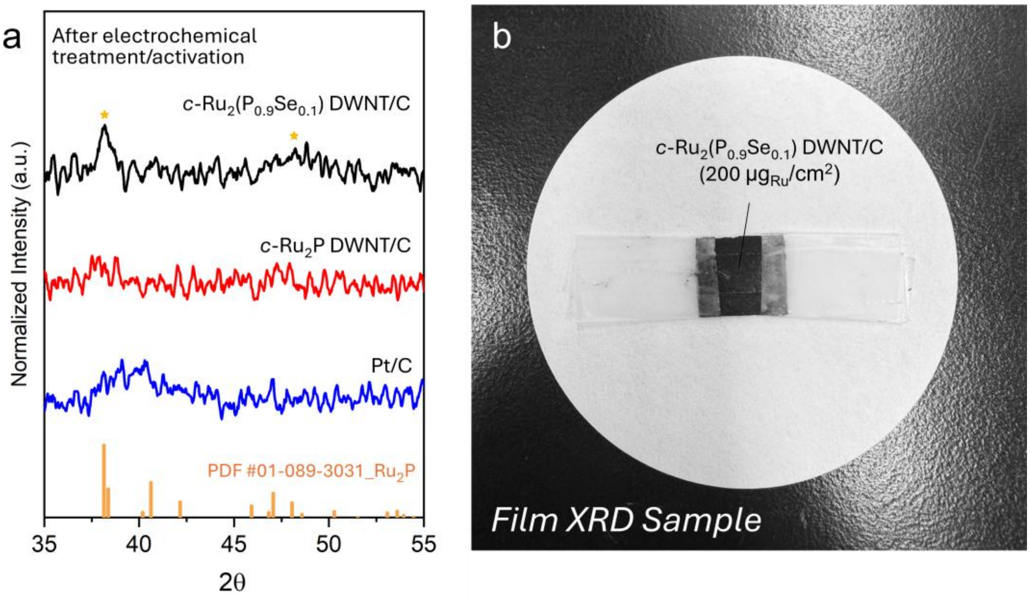

Figure S22. (a) XRD patterns of *c*–$Ru_2(P_{0.9}Se_{0.1})$ DWNT/C, *c*–Ru_2P DWNT/C, and Pt/C after electrochemical treatment/activation. (b) Carbon paper deposited catalysts after electrochemical treatment/activation was prepared for XRD analysis.

Note S3: To measure the XRD patterns of each catalyst after CV cycling, the prepared ink was applied onto carbon paper (1 cm^2) and subjected to electrochemical treatment. The amount of noble metal on the carbon electrode was 200 $\mu g_{Ru(Pt)}$ cm^{-2} for all catalysts. The XRD analysis of thin-film samples can produce significant noise due to contamination from the carbon paper and solvents (ex. DI water, IPA, electrolyte, etc.) compared to Powder-XRD analysis. [3]

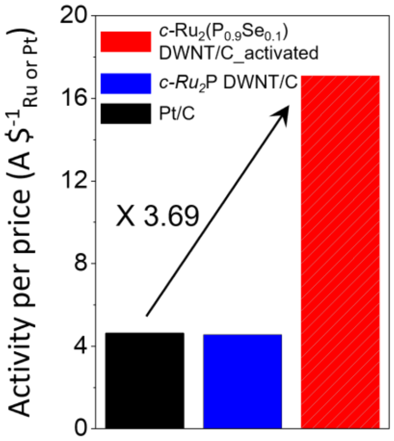

Figure S23. Price activity calculated at -0.02 V for activated *c*–$Ru_2(P_{0.9}Se_{0.1})$ DWNT/C, *c*–Ru_2P DWNT/C, and Pt/C based on the ICP-OES results.

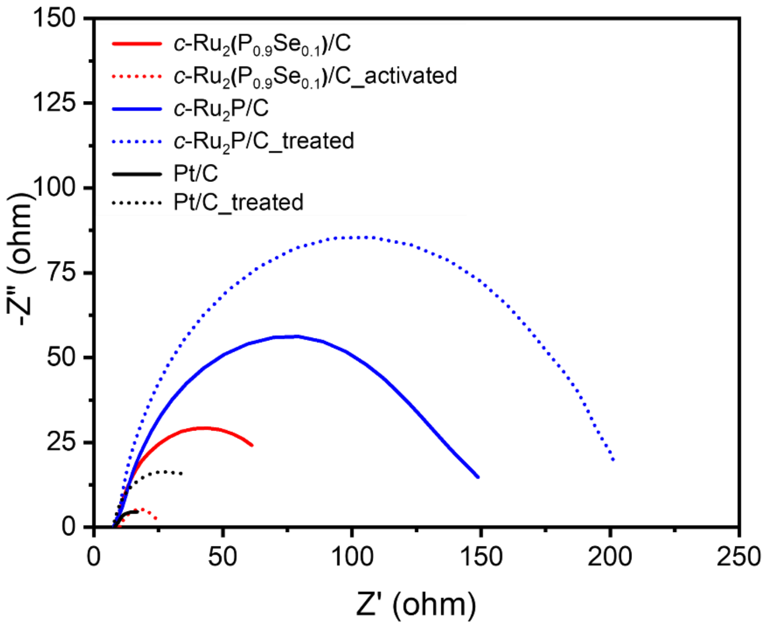

Figure S24. (a) Nyquist plots of the catalysts before and after electrochemical treatment/activation.

Note S4: The electrochemical impedance spectroscopy (EIS) spectra for activated *c*–$Ru_2(P_{0.9}Se_{0.1})$ DWNT/C, *c*–Ru_2P DWNT/C, and Pt/C show a semicircle, which indicates charge transfer resistance during the HER. The charge resistance (R_{ct}) value of the *c*–$Ru_2(P_{0.9}Se_{0.1})$ DWNT/C was lowered after activation process as small as Pt/C catalyst.

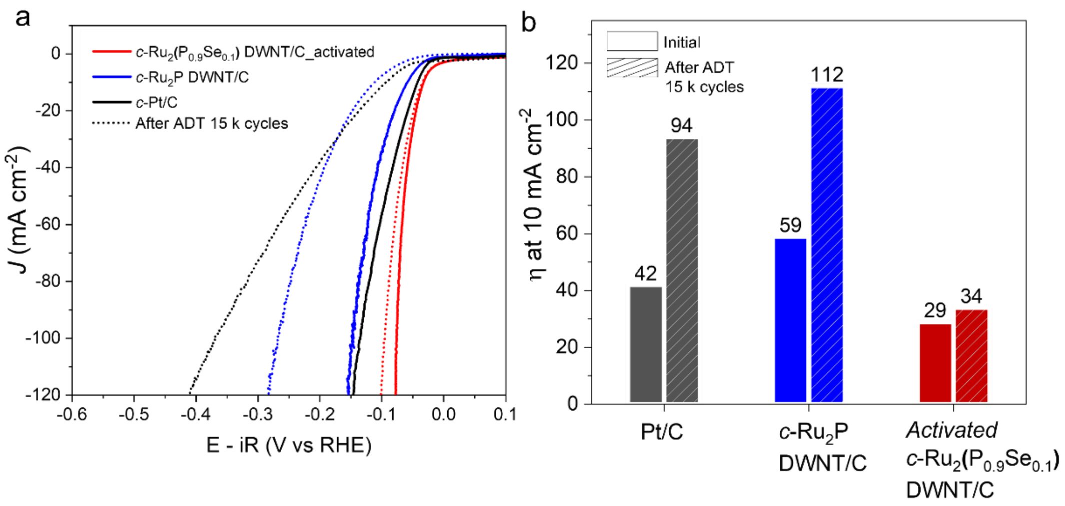

Figure S25. HER performances of the treated catalysts after accelerated durability test (ADT) for activated *c*–$Ru_2(P_{0.9}Se_{0.1})$ DWNT/C, *c*–Ru_2P DWNT/C, and Pt/C. (a) *iR*–corrected HER polarization curves of the catalysts before and after ADT 15 K cycles. (b) Overpotentials of catalysts derived from HER polarization curves at 10 mA cm^{-2}.

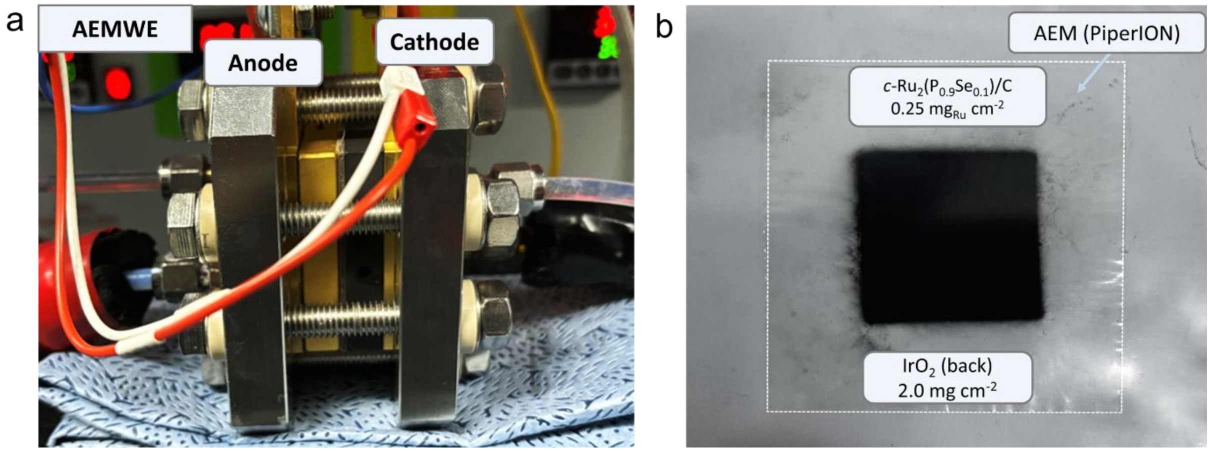

Figure S26. (a) A digital photograph of the operating AEMWE. (b) MEA fabricated form *c*-$Ru_2(P_{0.9}Se_{0.1})$ DWNT/C at the cathode (front, 0.25 mg_{Ru} cm^{-2}) and IrO_2 at anode (2.0 mg cm^{-2}), which were separated by a PiperION AEM.

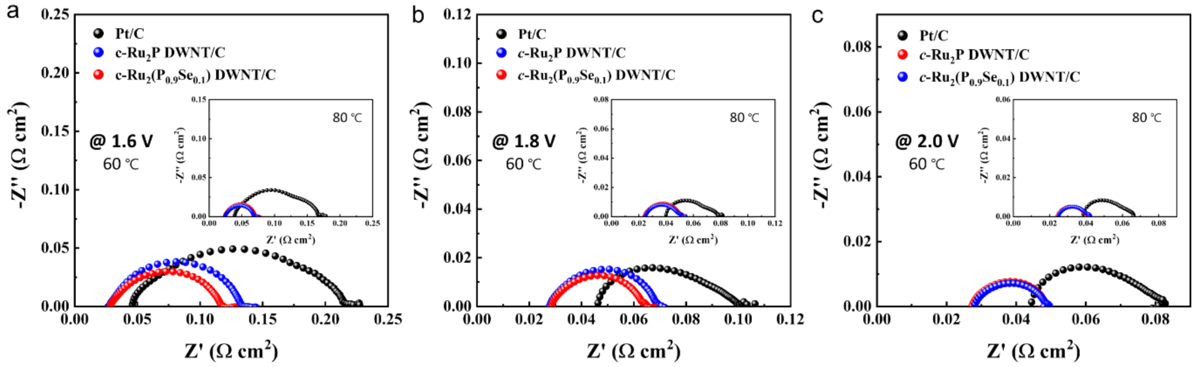

Figure S27. Electrochemical impedance spectroscopy (EIS) spectrum of electrolysis cells utilizing Pt/C, *c*-Ru_2P DWNT/C, and *c*-$Ru_2(P_{0.9}Se_{0.1})$ DWNT/C at three different voltages and different operating temperatures of 60 and 80 °C. (a) 1.6 V, (b) 1.8 V, (c) 2.0 V

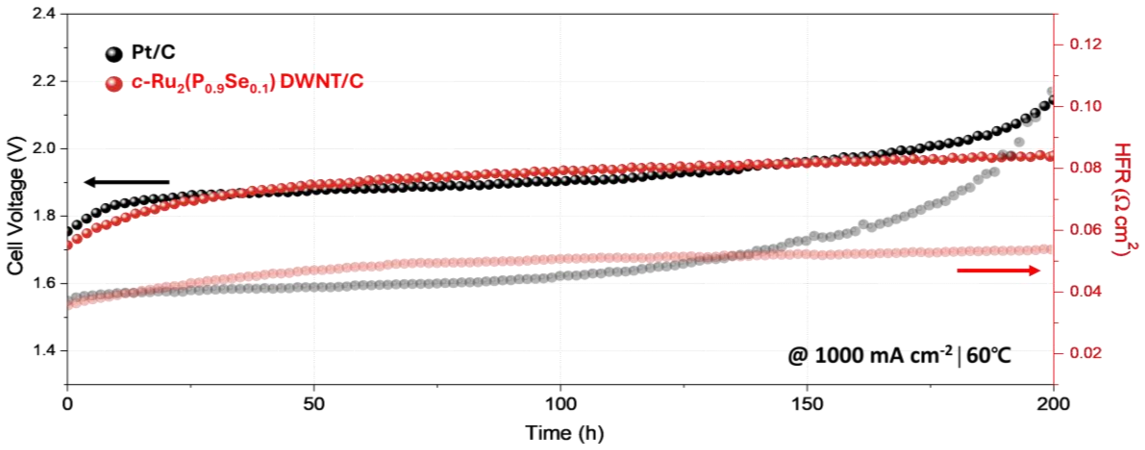

Figure S28. Comparison of durability cell voltage-time plots for the AEMWE based on Pt/C and *c*-$Ru_2(P_{0.9}Se_{0.1})$ DWNT/C at a constant current density of 1000 mA cm^{-2} at 60 °C.

Note S4. Computational hydrogen electrode model (CHE) for estimating HER activity.

Conventionally, the following reaction expresses the hydrogen evolution reaction (HER) in acidic conditions. [4-5]

$$H^+ + e^- \rightarrow 0.5H_2 \qquad \text{Eq. S1}$$

Initially, the adsorption of H-* on the surface as a hydrogen atom is defined as the Volmer step. Then, there are two routes, either the Heyrovsky step or the Tafel step for the evolution of H_2 (the * and H* denote the active site and adsorbed hydrogen atom on the surface of the catalyst).

$$\text{Volmer step: } H^+ + e^- + * \rightarrow H^* \qquad \text{Eq. S2}$$

$$\text{Heyrovsky step: } H^+ + H^* \rightarrow H_2 + * \qquad \text{Eq. S3}$$

$$\text{Tafel step: } H^* + H^* \rightarrow H_2 + * \qquad \text{Eq. S4}$$

For a reasonable DFT simulation to describe the HER catalytic activity, we employed the computational hydrogen electrode (CHE) model proposed by Noskov et al, [5] which treats the pair energy consisting of a proton and an electron as calculated by the half of the H_2 (g) molecule based on the following equation (applied potential (U) = 0 V, pH =0, p = 1 atm, T = 298.15 K).

$$G(H^+ + e^-) = G(0.5H_2), \qquad \Delta G^0 = 0\ eV \qquad \text{Eq. S5}$$

The CHE model with its equilibrium potentials is related to the thermodynamics of the reaction. Notably, when the adsorbate is adsorbed to the surface, Gibbs free energy (ΔG_{ads^*}) is defined as

$$\Delta G_{ads^*} = \Delta E_{ads^*} + \Delta ZPE - T\Delta S + \Delta G_U + \Delta G_{pH} \qquad \text{Eq. S6}$$

where ΔE_{ads^*} is the adsorption energy, ΔZPE is the change of zero-point energy after H* to the surface, ΔS is also the change of entropy with temperature (T = 298.15 K), [6] ΔG_U is correction term related to applied bias, ΔG_{pH} is an experimental term to consider proton concentration, which described as $\Delta G_{pH} = 2.303 * k_B T * pH$ *(or* $0.059 * pH$*)*, and k_B is the Boltzmann constant. [7] The adsorption energy is also defined as

$$\Delta E_{ads^*} = E_{ads^*} - E_* - E_{ads} \qquad \text{Eq. S7}$$

where E_{ads^*} is the total energy of the adsorbate adsorbed on the active site (*) of surface, E_* is the total energy of the target structure, E_{ads} is the total energy of adsorbate.

Especially, when the HER occurs in the alkaline condition, the Volmer and Heyrovsky steps are described with water molecules by following equations. [4]

$$\text{Volmer step: } H_2O + e^- + * \rightarrow H^* + OH^- \qquad \text{Eq. S8}$$

$$\text{Heyrovsky step: } H_2O + H^* + e^- \rightarrow H_2 + OH^- \qquad \text{Eq. S9}$$

To describe the alkaline HER activity initiated by H_2O, we drive a free energy relationship based on the CHE model. In the standard condition, the alkaline HER is derived as follows,

$$H_2O(l) + e^- \rightarrow 0.5H_2 + OH^-,\ (E^0 = -0.828\,V) \qquad \text{Eq. S10}$$

and the standard reduction potential (E^0) is -0.828 V at T = 298.15 K. In terms of chemical potentials, the equation is expressed as follows,

$$\mu_{H_2O(g)} + \mu_{e^-} = 0.5\mu_{H_2(g)} + \mu_{OH^-} \qquad \text{Eq. S11}$$

$$\mu_{e^-} - \mu_{OH^-} = 0.5\mu_{H_2(g)} - \mu_{H_2O(l)} \qquad \text{Eq. S12}$$

On the left side in Eq. S12, the μ_{e^-} and μ_{OH^-} could be derived further as follows,

$$\mu_{e^-} = \mu_{e^-}^0 - eU \qquad \text{Eq. S13}$$

$$\mu_{OH^-} = \mu_{OH^-}^0 + k_B T ln\alpha_{OH^-} \qquad \text{Eq. S14}$$

, where eU represents the shift in electron energy by an applied bias and $\mu_{e^-}^0$, $\mu_{OH^-}^0$ represents the chemical potentials of the electron, and hydroxide at standard conditions ($\alpha_{OH^-} = 1$, T = 298.15 K). Therefore, we can derive the following equations,

$$\mu_{e^-} - \mu_{OH^-} = ({\mu^0}_{e^-} - eU) - (\mu_{OH^-}^0 + k_B T ln\alpha_{OH^-}) \qquad \text{Eq. S15}$$

$$\mu_{e^-} - \mu_{OH^-} = {\mu^0}_{e^-} - \mu_{OH^-}^0 - eU = 0.5\mu_{H_2(g)} - \mu_{H_2O(l)} \qquad \text{Eq. S16}$$

at standard and equilibrium conditions ($\alpha_{OH^-} = 1$, T = 298.15 K, and U = E^0 = -0.828 V).

In the case of the right side in Eq. S12, the $\mu_{H_2O(l)}$ and $\mu_{H_2(g)}$ can be calculated from the approximations proposed by Noskov et al., where $\mu_{H_2O(l)}$ is equal to the $\mu_{H_2O(g)}$, at T = 298.15 K and 0.035 bars. [8] The $\mu_{H_2O(l)}$ is derived from the following equation,

$$\mu_{H_2O(l)} = \mu_{H_2O(g)} = E_{DFT}^{H_2O(g)} + ZPE_{H_2O(g)} - TS_{H_2O(g)}^0 \qquad \text{Eq. S17}$$

Moreover, the $\mu_{H_2(g)}$ is also derived from a similar approach.

$$\mu_{H_2(g)} = E_{DFT}^{H_2(g)} + ZPE_{H_2(g)} - TS_{H_2(g)}^0 \qquad \text{Eq. S18}$$

Therefore, the Eq. S12 can be written as:

$$\mu_{e^-} - \mu_{OH^-} = 0.5\mu_{H_2(g)} - \mu_{H_2O(g)} \qquad \text{Eq. S19}$$

Finally, we can obtain a value of 10.876 eV using Eq. S12, S17, and S19. All calculated ΔE_{DFT}, ΔZPE, and $T\Delta S$ values are described in Table S4-5.

Then, the free energies of each intermediate ($G_{H_2O^*}$, G_{H^*/OH^*}, and G_{H^*}) on the active site (*) of the catalyst surface could be calculated by following steps.

Step 1: $H_2O(g)$ + * → H_2O*

The first step is the adsorption of $H_2O(g)$ on an active site without the release of an electron:

$$\Delta G_1 = G_{H_2O*} - G_* - \mu_{H_2O(g)} \quad \text{Eq. S20}$$

, where respectively and could be expressed by DFT simulated total energies:

$$G_{H_2O*} = E_{DFT}^{H_2O^*} + ZPE_{H_2O^*} - TS_{H_2O^*}^{0} \quad \text{Eq. S21}$$

$$G_* = E_{DFT}^{*} \quad \text{Eq. S22}$$

Replacing Eq. S17, S21 and S22 in Eq. S20 we get:

$$\Delta G_1 = \left(E_{DFT}^{H_2O^*} - E_{DFT}^{*} - E_{DFT}^{H_2O(g)}\right) + \Delta ZPE - T\Delta S \quad \text{Eq. S23}$$

Step 2: H_2O* → H* + OH*

The second step is the dissociation of the H_2O* species to H* and OH* without the release of an electron:

$$\Delta G_2 = G_{H^*/OH^*} - G_{H_2O(g)} \quad \text{Eq. S24}$$

The relation for G_{H^*/OH^*} in terms of DFT energies is like the relation for Eq. S25. Replacing again the same equations as in the case for the first step in Eq. S28, we get:

$$\Delta G_2 = \left(E_{DFT}^{H^*/OH^*} - E_{DFT}^{H_2O^*}\right) + \Delta ZPE - T\Delta S \quad \text{Eq. S25}$$

Step 3: (H*/OH*) + e^- → H* + OH^-

The third step is represented by H* adsorption energy on the active site (*) with a release of an electron by OH^- desorption:

$$\Delta G_3 = G_{H^*} + \mu_{OH^-} - G_{H^*/OH^*} - \mu_{e^-} \quad \text{Eq. S26}$$

The relation in terms of DFT energies is also like the relation for Eq. S25. The same equations are replaced gradually in Eq. S26 as follows:

$$\Delta G_3 = \left(E_{DFT}^{H^*} - E_{DFT}^{H^*/OH^*}\right) + \Delta ZPE - T\Delta S - 10.876 \quad \text{Eq. S27}$$

Based on successfully derived reaction free energies (ΔG_1, ΔG_2, and ΔG_3), we calculated the binding free energies ($\Delta G_{H_2O^*}$, $\Delta G_{H^*/OH^*}$, and ΔG_{H^*}) until Volmer step of alkaline HER. [9]

Note S5. Structural design and OH* binding detail for Ru_2P-based catalysts

We constructed surface structures by cleaving the Ru_2P crystal structure along (020) index. We applied a vacuum space of 15 Å in the z-direction as a slab model to avoid interlayer interactions within the periodic boundary conditions and then fixed the two atomic layers at the bottom to represent the bulk. Surface energy (σ) was calculated using the following equation:

$$\sigma = (E_{Slab} - n * E_{Bulk})/(2 * \mathrm{A}) \qquad \text{Eq. S28}$$

, where E_{Slab} is the total energy of the fully relaxed (020) surface slab model, n is the number of bulk units to fit the stoichiometry of the surface slab model, E_{Bulk} is the total energy of Ru_2P bulk structures, and A is the surface area.

To investigate stably synthesized Se-doped $Ru_2P(020)$ catalyst by anion exchange process on the Ru_2P surface, we calculated the Se substitution reaction energy (E_{sub}) on the P to Se substituted $Ru_2P(020)$ surface. Based on the $Ru_2P(020)$ surface having $Ru_{80}P_{40}$ chemical formula, E_{sub} was calculated by the following equation:

$$E_{sub} = E_{Ru_{80}(P_{40-x}Se_x)} + xE_P - E_{Ru_{80}P_{40}} - xE_{Se})/x \;\; (4 \le x \le 8) \qquad \text{Eq. S29}$$

, where $E_{Ru_{80}(P_{40-x}Se_x)}$ is the total energy of Se substituted $Ru_2P(020)$ surface, E_P (or E_{Se}) are the total energy of bulk P (or Se), and $E_{Ru_{80}P_{40}}$ is the total energy of $Ru_2P(020)$ surface, x is the number of substituted Se atoms.

Especially, for the simultaneous consideration of n hydroxide adsorptions on Ru_2P-based catalyst (where n represents the number of adsorbed hydroxides in the system), we employed the integral hydroxides adsorption energy ($\Delta E_{OH^*}^{Int}$) of the multi-hydroxides adsorbed Ru_2P-based catalyst, expressed the following equation.[10-12]

$$\Delta E_{OH^*}^{Int} = (E_{Catalyst}^{OH(n)} - E_{Catalyst} - (nE_{H_2O} - \frac{n}{2}E_{H_2})/n \;\; (n \ge 1) \qquad \text{Eq. S30}$$

$$\Delta G_{OH^*}^{Int} = \Delta E_{OH^*}^{Int} + \Delta ZPE - T\Delta S \qquad \text{Eq. S31}$$

, where $E_{Catalyst}^{OH(n)}$ is the total energy of n hydroxide adsorbed Ru_2P-based surface structure, $E_{Catlayst}$ is the total energy of Ru_2P-based surface structure, and $\Delta G_{OH^*}^{Int}$ is the integral adsorption free energy.

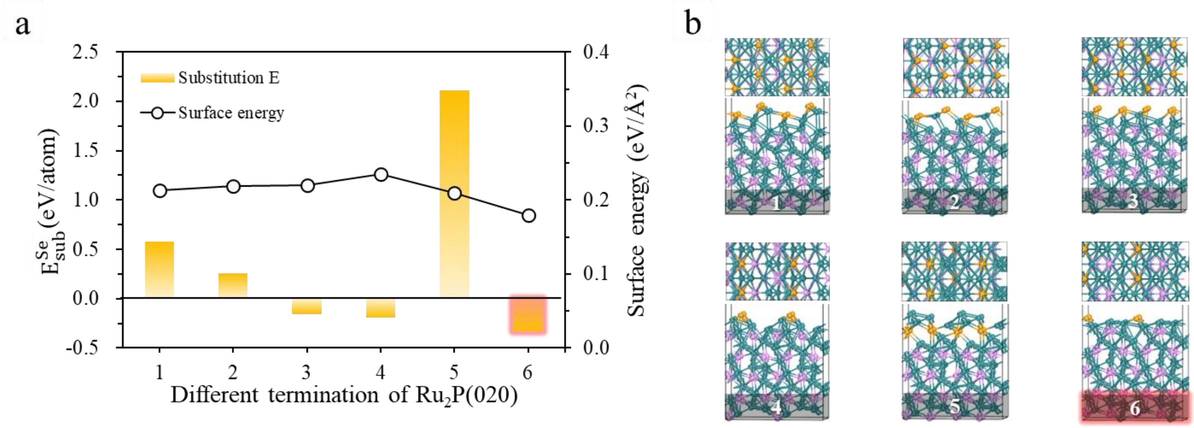

Figure S29. (a) Calculated Se substitution energy (E_{sub}^{Se}) and surface energy for various possible surface structures (1 to 6) of the $Ru_2P(020)$ structure and (b) top and side views of each structure.

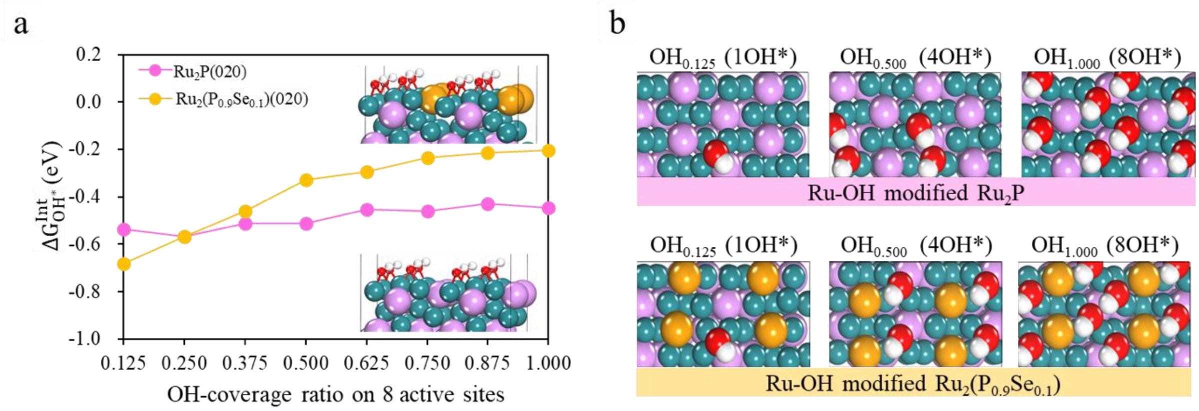

Figure S30. (a) Integral OH adsorption free energy ($\Delta G_{OH^*}^{Int}$) on Ru_2P(020) and $Ru_2(P_{0.9}Se_{0.1})$(020) structures considering OH*-coverage ratio. (b) top view of modified Ru_2P(020) and $Ru_2(P_{0.9}Se_{0.1})$(020) structures.

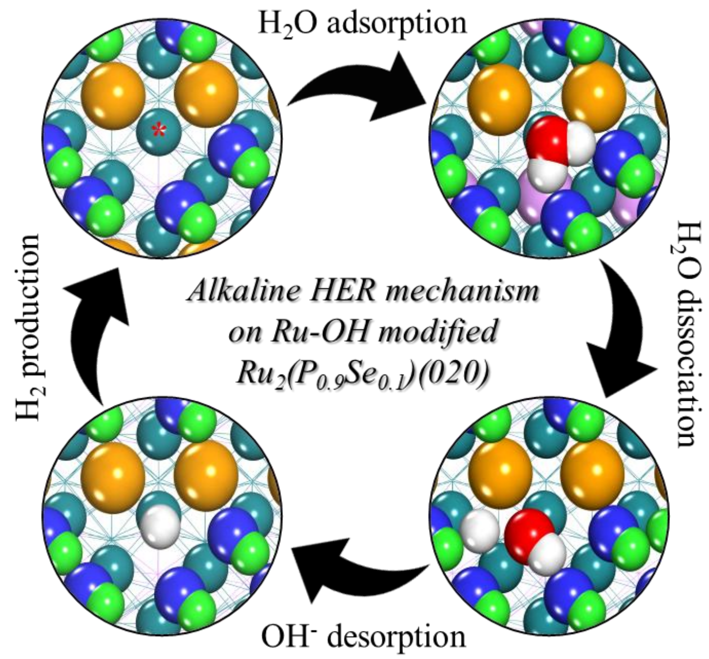

Figure S31. Alkaline HER mechanism on Ru-OH modified $Ru_2(P_{0.9}Se_{0.1})(020)$ surface. The colors orange, green, fluorescent green, blue, red, and white correspond to Se, Ru, pre-adsorbed H, pre-adsorbed O, O, and H respectively.

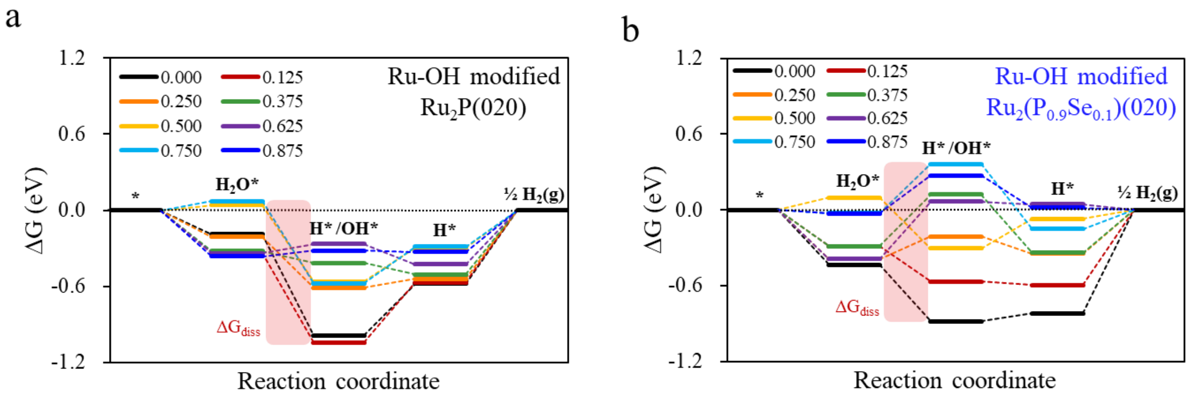

Figure S32. HER free energy diagrams (FEDs) of the Ru-OH modified (a) $Ru_2P(020)$ and (b) $Ru_2(P_{0.9}Se_{0.1})(020)$ surface structures in an alkaline environment.

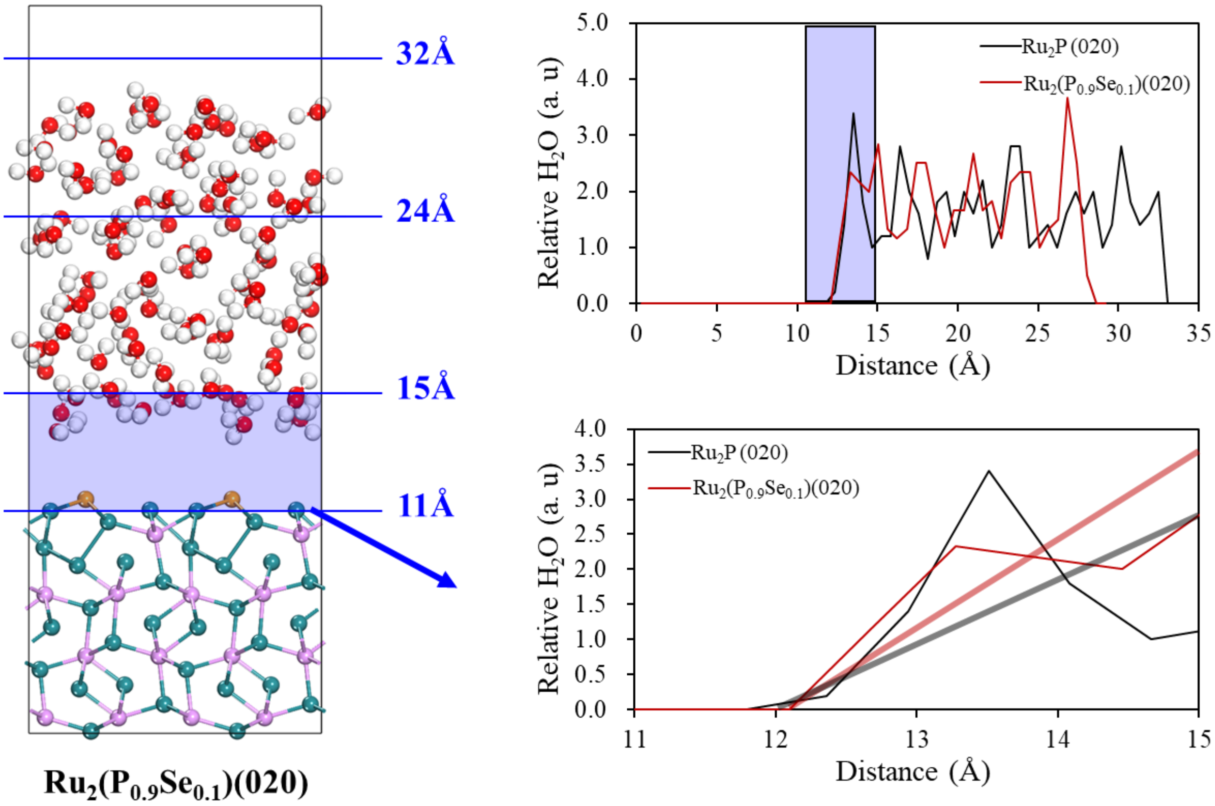

Figure S33. (a) H_2O containing $Ru_2(P_{0.9}Se_{0.1})(020)$ surface structure for water penetration by molecular dynamics (MD) simulation, the relative H_2O concentration according to $Ru_2P(020)$ and $Ru_2(P_{0.9}Se_{0.1})(020)$ in (b) the large distance range, and (c) low distance range.

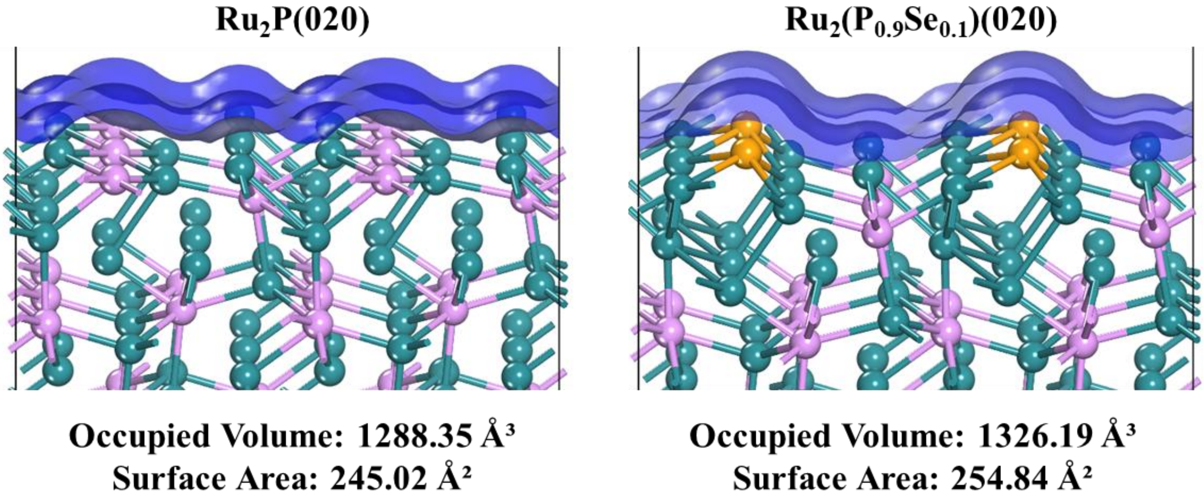

Figure S34. Estimated Conolly volume of $Ru_2P(020)$ and $Ru_2(P_{0.9}Se_{0.1})(020)$ structures.

Table S1. Structural parameters of references (Ru foil and RuO_2) and catalysts from the EXAFS fitting for Ru K–edges.

Material	Shell	CN	R (Å)	σ^2 (Å^2)	ΔE_0 (eV)	R factor
Ru	Ru–Ru1	6.0	2.64	0.00233	–0.051	0.00864
	Ru–Ru2	6.0	2.70	0.00186	–0.051	
RuO_2	Ru–O1	2.0	1.90	0.00872	7.468	0.00756
	Ru–O2	4.0	1.96	0.00066	6.616	
c–Ru_2P DWNT	Ru–P	5.8	2.24	0.01527	–7.259	0.00876
	Ru–Ru	4.3	2.78	0.01002	2.255	
c–$Ru_2(P_{0.9}Se_{0.1})$ DWNT	Ru–Se	4.8	2.22	0.00644	–0.581	0.00212
	Ru–P	4.4	2.27	0.01484	–0.004	
	Ru–Ru	2.3	2.78	0.00377	4.481	

CN: coordination numbers; R: bond distance; σ^2: Debye–Waller factors; ΔE_0: the inner potential correction. R factor: goodness of it. S_0^2 was set to 0.684, according to the

experimental EXAFS fit of Ru foil reference by fixing CN as the known crystallographic value.

Table S2. Summary of some recently reported representative HER electrocatalysts in alkaline electrolytes.

Catalyst	Catalyst loading (mg cm^{-2})	η (mV) @ 10 mA cm^{-2}	Tafel slope (mV dec^{-1})	Electrolyte	Ref.
Activated c–$Ru_2(P_{0.9}Se_{0.1})$	0.100	29	32	*1.0 M KOH*	*This study*
$NI_{1.5}Co_{1.4}P$@Ru	0.280	52	42	*1.0 M KOH*	[13]
$Ru_{0.33}Se$@TNA	0.200	57	50	*1.0 M KOH*	[14]
$Cu_{2-x}S$@RuNPs	0.230	82	48	*1.0 M KOH*	[15]
Ru–MoS_2/CNT	1.000	50	62	*1.0 M KOH*	[16]
RuCo@NC	0.273	45	66	*1.0 M KOH*	[17]
Ru@Co/N–CNTs	–	48	33	*1.0 M KOH*	[18]
RuP_x/NPC	0.195	74	70	*1.0 M KOH*	[19]
RuP_2@NPC	1.000	52	69	*1.0 M KOH*	[20]
Ru@NG–4	0.857	40	76	*1.0 M KOH*	[21]
Ru–Ni@Ni_2P–HNRs	1.000	31	41	*1.0 M KOH*	[22]
NiRu@N–C	0.273	32	64	*1.0 M KOH*	[23]
RuCoP	0.300	23	37	*1.0 M KOH*	[24]
Ru–MoO_2	0.285	29	31	*1.0 M KOH*	[25]
RuP–475	0.348	22	36	*1.0 M KOH*	[26]

Table S3. Comparison of the state-of-art AEMWEs in current research

Cathode /catalyst	Anode /catalyst	AEM	Anode /cathode electrolyte	Temp	AEMWE current density	Durability	Ref
***c*-$Ru_2(P_{0.9}Se_{0.1})$ DWNT/C**	**IrO_2**	**PiperION**	**A/C 1 M KOH**	**80 °C**	**10.31 A cm^{-2} @ 2.0 V**	**200 h @ 1.0 A cm^{-2}**	**This work**
Pt/C	Ir black	Aemion™	A/C 1 M KOH	50 °C	0.5 A cm^{-2} @ 1.68 V	1000 min @ 0.5 A cm^{-2}	[27]
Pt/C	IrO_2	PFTP-13	A: 1 M KOH	80 °C	7.68 A cm^{-2} @ 2.0 V	1100 h @ 0.5 A cm^{-2}	[28]
Pt/C	IrO_2	PFTP-8	A: 1 M KOH	80 °C	4.99 A cm^{-2} @ 2.0 V	-	[28]
Pt/C	IrO_2	Orion TM1™	A/C 1 M KOH	70 °C	2.75 A cm^{-2} @ 1.9 V	50 h @ 0.5 A cm^{-2}	[29]
NiMo	Ni	PBI+KOH	A/C 24% KOH	80 °C	2.0 A cm^{-2} @ 1.85 V	-	[30]
Ni-Fe	Ni-Fe	PFTP-Sustainion®	A: 1 M KOH	60 °C	0.62 A cm^{-2} @ 2.0 V	140 h @ 0.5 A cm^{-2}	[28]
Fe-Ni-Mo	Ni-Mo	PFTP-Sustainion®	A/C 1 M KOH	80 °C	1.0 A cm^{-2} @ 1.57 V	-	[31]
Pt/C	NiFeV LDH	X37-50 Grade T	A/C 1 M KOH	50 °C	2.89 A cm^{-2} @ 1.9 V	100 h @ 0.5 A cm^{-2}	[32]
Pt/C	NiFeOOH	FAA-3-50	A/C 1 M KOH	60 °C	3.6 A cm^{-2} @ 1.9 V	200 h @ 0.1 A cm^{-2}	[33]
Pt/C	Co_3O_4	HTMA-DAPP	1% K_2CO_3	50 °C	-	750 h @ 0.5 A cm^{-2}	[34]
Pt/C	IrO_2	QPC-TMA	A/C 1 M KOH	70 °C	4.5 A cm^{-2} @ 2.0 V	2.7 h @ 1.67 V	[35]
Pt/C	IrO_2	QPC-TMA	A: CH_2O	70 °C	0.4 A cm^{-2} @ 2.0 V	-	[35]
Pt/C	Fe_xNi_yOOH-20F	PAP-TP-85	A/C 1 M KOH	80 °C	1.5 A cm^{-2} @ 1.74 V	-	[36]
Pt/C	Fe_xNi_yOOH-20F	AP-TP-85	A/C H_2O	80 °C	1.02 A cm^{-2} @ 1.8 V	160 h @ 0.2 A cm^{-2}	[36]
Pt/C	IrO_2	PIS	A/C 0.5 M KOH	80 °C	0.55 A cm^{-2} @ 2.0 V	80 h @ 1.8 V	[37]
Pt/C	IrO_2	A201	A/C 15% KOH	50 °C	0.4 A cm^{-2} @ 1.8 V	-	[38]
Pt-Ru/C	Ni-Fe	HTMA-DAPP	A/C 1 M NaOH	85 °C	5.3 A cm^{-2} @ 1.85 V	14 h @ 0.2 A cm^{-2}	[39]
Pt-Ru/C	IrO_2	HTMA-DAPP	A/C H_2O	60 °C	0.4 A cm^{-2} @ 2.0 V	-	[40]
Pt-Ru/C	IrO_2	SES-TMA	A: H_2O	60 °C	-	100 h @ 0.1 A cm^{-2}	[34]
$PtRuP_2$	IrO_2	PiperION	A/C 1 M KOH	80 °C	9.4 A cm^{-2} @ 2.0 V	270 h @ 1.0 A cm^{-2}	[41]

State-of-art PEMWEs							
Pt/C	IrNS	Nafion 117	H_2O	80 °C	4 A cm^{-2} @ 2.02 V	90 h @ 3 A cm^{-2}	[42]
Pt/C	PtCo-RuO_2/C	Nafion 212	H_2O	80 °C	4.4 A cm^{-2} @ 2.0 V	24 h @ 1 A cm^{-2}	[43]
Pt/C	Fe_2N @ EIROF	Nafion 115	A: H_2O	80 °C	4.5 A cm^{-2} @ 1.9 V	120 h @ 2 A cm^{-2}	[44]
Pt/C	Sr_2MIrO_6	Nafion 212	A/C 0.5 M H_2SO_4	80 °C	3.4 A cm^{-2} @ 2.0 V	450 h @ 2 A cm^{-2}	[45]
Pt/C	IrO_2	Nafion 212	H_2O	80 °C	6 A cm^{-2} @ 2.0 V	-	[46]

Table S4 Calculated thermodynamic values for free energy diagram (FED) of $Ru_2P(020)$ surface structures, adsorption binding energies ($\Delta E_{H_2O^*}$, $\Delta E_{H^*/OH^*}$, and ΔE_{H^*}), zero-point energy (ZPE) and entropy contribution TΔS of intermediates (H_2O^*, H*/OH*, and H*).

Surface type	OH* ratio	$\Delta E_{H_2O^*}$ (eV)	$\Delta E_{H^*/OH^*}$ (eV)	ΔE_{H^*} (eV)	H_2O^*	H*/OH*	H*
					ZPE/TΔS (eV)		
$Ru_2P(020)$	0.000	-0.88	-1.52	-0.81	0.69/ 0.10	0.52/ 0.15	0.17/ 0.01
	0.125	-1.03	-1.57	-0.80			
	0.250	-0.90	-1.14	-0.77			
	0.375	-1.01	-0.95	-0.73			
	0.500	-0.65	-1.09	-0.52			
	0.625	-1.03	-0.80	-0.65			
	0.750	-0.62	-1.11	-0.51			
	0.875	-1.05	-0.85	-0.55			

Table S5 Calculated thermodynamic values for free energy diagram (FED) of $Ru_2(P_{0.9}Se_{0.1})$ (020) surface structures, adsorption binding energies ($\Delta E_{H_2O^*}$, $\Delta E_{H^*/OH^*}$, and ΔE_{H^*}), zero-point energy (ZPE) and entropy contribution TΔS of intermediates (H_2O*, H*/OH*, and H*).

Surface type	OH* ratio	$\Delta E_{H_2O^*}$ (eV)	$\Delta E_{H^*/OH^*}$ (eV)	ΔE_{H^*} (eV)	H_2O*	H*/OH*	H*
					ZPE/TΔS (eV)		
$Ru_2(P_{0.9}Se_{0.1})$ (020)	0.000	-1.07	-0.88	-0.82	0.67/ 0.14	0.51/ 0.10	0.17/ 0.01
	0.125	-0.92	-0.57	-0.59			
	0.250	-1.03	-0.21	-0.35			
	0.375	-0.93	0.12	-0.34			
	0.500	-0.54	-0.31	-0.07			
	0.625	-1.03	0.06	0.04			
	0.750	-0.66	0.35	-0.15			
	0.875	-0.67	0.27	0.02			

Reference

[1] S. Jeong, C. Song, J. Kim, Y. Lee, M. H. Kim, *J. Alloys Compd.* **2023**, *947*, 169649.
[2] J.-C. Kim, C. W. Lee, D.-W. Kim, *J. Mater. Chem. A* **2020**, *8*, 5655.
[3] T. Ryan, *J. Chem. Educ.* **2001**, *78*, 613.
[4] J. Kibsgaard, T. F. Jaramillo, *Angew. Chem., Int. Ed.* **2014**, *53*, 14433.
[5] C. C. L. McCrory, S. Jung, I. M. Ferrer, S. M. Chatman, J. C. Peters, T. F. Jaramillo, *J. Am. Chem. Soc.* **2015**, *137*, 4347.
[6] J. Kibsgaard, C. Tsai, K. Chan, J. D. Benck, J. K. Nørskov, F. Abild-Pedersen, T. F. Jaramillo, *Energy Environ. Sci.* **2015**, *8*, 3022.
[7] P. Quaino, F. Juarez, E. Santos, W. Schmickler, *Beilstein J. Nanotechnol.* **2014**, *5*, 846.
[8] J. K. Nørskov, T. Bligaard, A. Logadottir, J. R. Kitchin, J. G. Chen, S. Pandelov, U. Stimming, *J. Electrochem. Soc.* **2005**, *152*, J23.
[9] Y. Bai, B. W. J. Chen, G. Peng, M. Mavrikakis, *Catal. Sci. Technol.* **2018**, *8*, 3321.
[10] I. C. Man, H.-Y. Su, F. Calle-Vallejo, H. A. Hansen, J. I. Martínez, N. G. Inoglu, J. Kitchin, T. F. Jaramillo, J. K. Nørskov, J. Rossmeisl, *ChemCatChem* **2011**, *3*, 1159.
[11] C. H. Lee, E. B. Nam, S. U. Lee, *J. Mater. Chem. A* **2019**, *7*, 22615.
[12] C. H. Lee, B. Jun, S. U. Lee, *RSC Adv.* **2017**, *7*, 27033.
[13] S. Liu, Q. Liu, Y. Lv, B. Chen, Q. Zhou, L. Wang, Q. Zheng, C. Che, C. Chen, *Chem. Commun.* **2017**, *53*, 13153.
[14] K. Wang, Q. Chen, Y. Hu, W. Wei, S. Wang, Q. Shen, P. Qu, *Small* **2018**, *14*, 1802132.
[15] D. Yoon, J. Lee, B. Seo, B. Kim, H. Baik, S. H. Joo, K. Lee, *Small* **2017**, *13*, 1700052.
[16] X. Zhang, F. Zhou, S. Zhang, Y. Liang, R. Wang, *Adv. Sci.* **2019**, *6*, 1900090.
[17] Y. Xu, Y. Li, S. Yin, H. Yu, H. Xue, X. Li, H. Wang, L. Wang, *Nanotechnology* **2018**, *29*, 225403.
[18] Z. Liu, X. Yang, G. Hu, L. Feng, *ACS Sustain. Chem. Eng.* **2020**, *8*, 9136.
[19] J.-Q. Chi, W.-K. Gao, J.-H. Lin, B. Dong, K.-L. Yan, J.-F. Qin, B. Liu, Y.-M. Chai, C.-G. Liu, *ChemSusChem* **2018**, *11*, 743.
[20] Z. Pu, I. S. Amiinu, Z. Kou, W. Li, S. Mu, *Angew. Chem., Int. Ed.* **2017**, *56*, 11559.
[21] B. K. Barman, D. Das, K. K. Nanda, *Sustain. Energy Fuels* **2017**, *1*, 1028.
[22] Y. Zuo, S. Bellani, G. Saleh, M. Ferri, D. V. Shinde, M. I. Zappia, J. Buha, R. Brescia, M. Prato, R. Pascazio, A. Annamalai, D. O. de Souza, L. De Trizio, I. Infante, F. Bonaccorso, L. Manna, *J. Am. Chem. Soc.* **2023**, *145*, 21419.
[23] Y. Xu, S. Yin, C. Li, K. Deng, H. Xue, X. Li, H. Wang, L. Wang, *J. Mater. Chem. A* **2018**, *6*, 1376.
[24] J. Xu, T. Liu, J. Li, B. Li, Y. Liu, B. Zhang, D. Xiong, I. Amorim, W. Li, L. Liu, *Energy Environ. Sci.* **2018**, *11*, 1819.
[25] P. Jiang, Y. Yang, R. Shi, G. Xia, J. Chen, J. Su, Q. Chen, *J. Mater. Chem. A* **2017**, *5*, 5475.
[26] Q. Chang, J. Ma, Y. Zhu, Z. Li, D. Xu, X. Duan, W. Peng, Y. Li, G. Zhang, F. Zhang, X. Fan, *ACS Sustain. Chem. Eng.* **2018**, *6*, 6388.
[27] P. Fortin, T. Khoza, X. Cao, S. Y. Martinsen, A. Oyarce Barnett, S. Holdcroft, *J. Power Sources* **2020**, *451*, 227814.
[28] N. Chen, S. Y. Paek, J. Y. Lee, J. H. Park, S. Y. Lee, Y. M. Lee, *Energy Environ. Sci.* **2021**, *14*, 6338.
[29] S. Y. Kang, J. E. Park, G. Y. Jang, O.-H. Kim, O. J. Kwon, Y.-H. Cho, Y.-E. Sung, *Int. J. Hydrogen Energy* **2022**, *47*, 9115.
[30] M. R. Kraglund, M. Carmo, G. Schiller, S. A. Ansar, D. Aili, E. Christensen, J. O. Jensen, *Energy Environ. Sci.* **2019**, *12*, 3313.

[31] P. Chen, X. Hu, *Adv. Energy Mater.* **2020**, *10*, 2002285.

[32] J. Lee, H. Jung, Y. S. Park, S. Woo, J. Yang, M. J. Jang, J. Jeong, N. Kwon, B. Lim, J. W. Han, S. M. Choi, *Small* **2021**, *17*, 2100639.

[33] J. E. Park, S. Park, M.-J. Kim, H. Shin, S. Y. Kang, Y.-H. Cho, Y.-E. Sung, *ACS Catal.* **2022**, *12*, 135.

[34] D. Li, A. R. Motz, C. Bae, C. Fujimoto, G. Yang, F.-Y. Zhang, K. E. Ayers, Y. S. Kim, *Energy Environ. Sci.* **2021**, *14*, 3393.

[35] M. S. Cha, J. E. Park, S. Kim, S.-H. Han, S.-H. Shin, S. H. Yang, T.-H. Kim, D. M. Yu, S. So, Y. T. Hong, S. J. Yoon, S.-G. Oh, S. Y. Kang, O.-H. Kim, H. S. Park, B. Bae, Y.-E. Sung, Y.-H. Cho, J. Y. Lee, *Energy Environ. Sci.* **2020**, *13*, 3633.

[36] J. Xiao, A. M. Oliveira, L. Wang, Y. Zhao, T. Wang, J. Wang, B. P. Setzler, Y. Yan, *ACS Catal.* **2021**, *11*, 264.

[37] X. Hu, Y. Huang, L. Liu, Q. Ju, X. Zhou, X. Qiao, Z. Zheng, N. Li, *J. Membr. Sci.* **2021**, *621*, 118964.

[38] J. Zhu, X. Meng, J. Zhao, Y. Jin, N. Yang, S. Zhang, J. Sunarso, S. Liu, *J. Membr. Sci.* **2017**, *535*, 143.

[39] D. Li, E. J. Park, W. Zhu, Q. Shi, Y. Zhou, H. Tian, Y. Lin, A. Serov, B. Zulevi, E. D. Baca, C. Fujimoto, H. T. Chung, Y. S. Kim, *Nat. Energy* **2020**, *5*, 378.

[40] J. Liu, Z. Kang, D. Li, M. Pak, S. M. Alia, C. Fujimoto, G. Bender, Y. S. Kim, A. Z. Weber, *J. Electrochem. Soc.* **2021**, *168*, 054522.

[41] Y. Hong, S. C. Cho, S. Kim, H. Jin, J. H. Seol, T. K. Lee, J.-k. Ryu, G. M. Tomboc, T. Kim, H. Baik, C. Choi, J. Jo, S. Jeong, E. Lee, Y. Jung, D. Ahn, Y.-T. Kim, S. J. Yoo, S. U. Lee, K. Lee, *Adv. Energy Mater.* **2024**, *14*, 2304269.

[42] Z. Xie, L. Ding, S. Yu, W. Wang, C. B. Capuano, A. Keane, K. Ayers, D. A. Cullen, H. M. Meyer, F.-Y. Zhang, *Appl. Catal. B Environ.* **2024**, *341*, 123298.

[43] H. Jin, S. Choi, G. J. Bang, T. Kwon, H. S. Kim, S. J. Lee, Y. Hong, D. W. Lee, H. S. Park, H. Baik, Y. Jung, S. J. Yoo, K. Lee, *Energy Environ. Sci.* **2022**, *15*, 1119.

[44] H.-Y. Jeong, J. Oh, G. S. Yi, H.-Y. Park, S. K. Cho, J. H. Jang, S. J. Yoo, H. S. Park, *Appl. Catal. B Environ.* **2023**, *330*, 122596.

[45] M. Retuerto, L. Pascual, J. Torrero, M. A. Salam, Á. Tolosana-Moranchel, D. Gianolio, P. Ferrer, P. Kayser, V. Wilke, S. Stiber, V. Celorrio, M. Mokthar, D. G. Sanchez, A. S. Gago, K. A. Friedrich, M. A. Peña, J. A. Alonso, S. Rojas, *Nat. Commun.* **2022**, *13*, 7935.

[46] M. Bernt, H. A. Gasteiger, *J. Electrochem. Soc.* **2016**, *163*, F3179.
